# Deep Learning Paradigm and Its Bias for Coronary Artery Wall Segmentation in Intravascular Ultrasound Scans: A Closer Look

**DOI:** 10.3390/jcdd10120485

**Published:** 2023-12-04

**Authors:** Vandana Kumari, Naresh Kumar, Sampath Kumar K, Ashish Kumar, Sanagala S. Skandha, Sanjay Saxena, Narendra N. Khanna, John R. Laird, Narpinder Singh, Mostafa M. Fouda, Luca Saba, Rajesh Singh, Jasjit S. Suri

**Affiliations:** 1 School of Computer Science and Engineering, Galgotias University, Greater Noida 201310, India; vandana.soni80@gmail.com (V.K.); ksampathkumara@gmail.com (S.K.K.); 2 Department of Applied Computational Science and Engineering, G L Bajaj Institute of Technology and Management, Greater Noida 201310, India; 3 School of CSET, Bennett University, Greater Noida 201310, India; ashish.gupta14d@gmail.com; 4 Department of CSE, CMR College of Engineering and Technology, Hyderabad 501401, India; sivaskandha@cmrcet.org; 5 Department of Computer Science and Engineering, IIT Bhubaneswar, Bhubaneswar 751003, India; sanjay@iiit-bh.ac.in; 6 Department of Cardiology, Indraprastha APOLLO Hospitals, New Delhi 110076, India; drnnkhanna@gmail.com; 7 Heart and Vascular Institute, Adventist Health St. Helena, St Helena, CA 94574, USA; lairdjr@ah.org; 8 Department of Food Science and Technology, Graphic Era, Deemed to be University, Dehradun 248002, India; narpinders@yahoo.com; 9 Department of Electrical and Computer Engineering, Idaho State University, Pocatello, ID 83209, USA; mfouda@ieee.org; 10 Department of Radiology, Azienda Ospedaliero Universitaria (A.O.U.), 09100 Cagliari, Italy; lucasabamd@gmail.com; 11 Department of Research and Innovation, Uttaranchal Institute of Technology, Uttaranchal University, Dehradun 248007, India; drrajeshsingh004@gmail.com; 12 Stroke Diagnostics and Monitoring Division, AtheroPoint™, Roseville, CA 95661, USA; 13 Department of Computer Science & Engineering, Graphic Era, Deemed to be University, Dehradun 248002, India; 14 Monitoring and Diagnosis Division, AtheroPoint™, Roseville, CA 95661, USA

**Keywords:** coronary artery disease, intravascular ultrasound, deep learning, UNet, wall segmentation, AI bias

## Abstract

Background and Motivation: Coronary artery disease (CAD) has the highest mortality rate; therefore, its diagnosis is vital. Intravascular ultrasound (IVUS) is a high-resolution imaging solution that can image coronary arteries, but the diagnosis software via wall segmentation and quantification has been evolving. In this study, a deep learning (DL) paradigm was explored along with its bias. Methods: Using a PRISMA model, 145 best UNet-based and non-UNet-based methods for wall segmentation were selected and analyzed for their characteristics and scientific and clinical validation. This study computed the coronary wall thickness by estimating the inner and outer borders of the coronary artery IVUS cross-sectional scans. Further, the review explored the bias in the DL system for the first time when it comes to wall segmentation in IVUS scans. Three bias methods, namely (i) ranking, (ii) radial, and (iii) regional area, were applied and compared using a Venn diagram. Finally, the study presented explainable AI (XAI) paradigms in the DL framework. Findings and Conclusions: UNet provides a powerful paradigm for the segmentation of coronary walls in IVUS scans due to its ability to extract automated features at different scales in encoders, reconstruct the segmented image using decoders, and embed the variants in skip connections. Most of the research was hampered by a lack of motivation for XAI and pruned AI (PAI) models. None of the UNet models met the criteria for bias-free design. For clinical assessment and settings, it is necessary to move from a paper-to-practice approach.

## 1. Introduction

**Figure 1:**
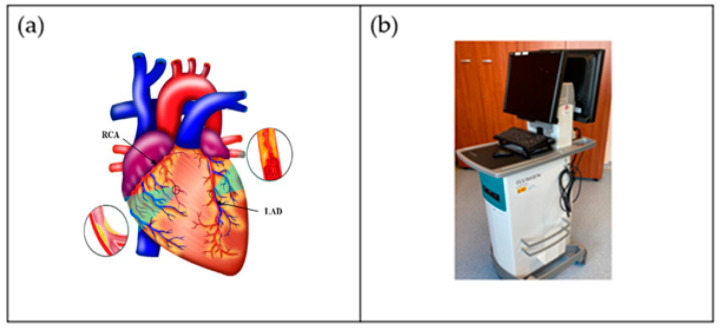
(**a**) Coronary arteries of the heart showing LAD (left anterior descending coronary artery) and RCA (right coronary artery) (Courtesy of AtheroPoint™, Roseville, CA, USA). (**b**) IVUS acquisition device (Courtesy of Dr. Alberto Boi and Luca Saba, University of Cagliari, Cagliari, Italy).

One of the world’s greatest contributors to mortality and morbidity is cardiovascular disease (CVD), which accounts for about 18 million deaths per year [1]. The primary two causes of CVD-related fatalities are coronary artery disease (CAD) and acute coronary syndrome (ACS) [2]. Generally speaking, CAD entails the shrinking of arteries as a result of the buildup of atherosclerotic plaque within their walls, resulting in coronary artery obstruction [3]. Aiming to enhance the diagnosis and treatment of heart disorders as well as lowering the fatality rate from CVD, significant advancements have been made in cardiovascular research and therapy in recent decades [4]. It is now possible to carry out a comprehensive qualitative and quantitative assessment of heart morphological structures as well as operations with the use of contemporary medical imaging techniques, including intravascular ultrasound (IVUS) [5,6,7,8,9], computed tomography (CT) [10], magnetic resonance imaging (MRI) [11,12,13], and ultrasound (US) [14,15], which assist identification, disease monitoring, surgical planning, and evaluation. An example of the coronary artery is shown in Figure 1a, while the IVUS acquisition device for the coronary vascular system is shown in Figure 1b.

The diagnosis of CAD is frequently made by coronary CT angiography (CCTA), which enables non-invasive measurement of the arterial lumen’s diameter and plaque localization [16,17,18,19,20]. However, radiologists presently manually assess the location and severity of the plaque(s) leading to the stenosis in CCTA pictures, which, in addition to being costly and time-consuming, is also susceptible to mistake and inaccuracy [21]. In order to develop computerized and accurate coronary artery stenosis as well as a plaque identification method, it is crucial that coronary arteries in CCTA pictures must be automatically segmented. The following factors, however, make automatic coronary artery segmentation for CCTA pictures particularly complicated. To begin with, coronary circulation has a complicated pattern, with several arteries of different thicknesses [22]. For perfect segmentation, some of the branches are even too thin. Additionally, individual differences in the structure of the coronary artery tree may be relevant. Second, other vascular organs that seem like the coronary arteries adjacent to the heart can be mistaken for them because of their similar appearance [23]. Third, the coronary arteries only make up a tiny fraction of the entire heart’s cells, and the methods for segmentation must consider this imbalance [24]. Additionally, several variables, including heart rate, the data reconstruction method, the quantity of the injected contrast agent, and radiation exposure, affect the quality of the pictures obtained during CT angiography [25]. Coronary artery segmentation, therefore, is more challenging due to low-resolution image quality. 

**Figure 2:**
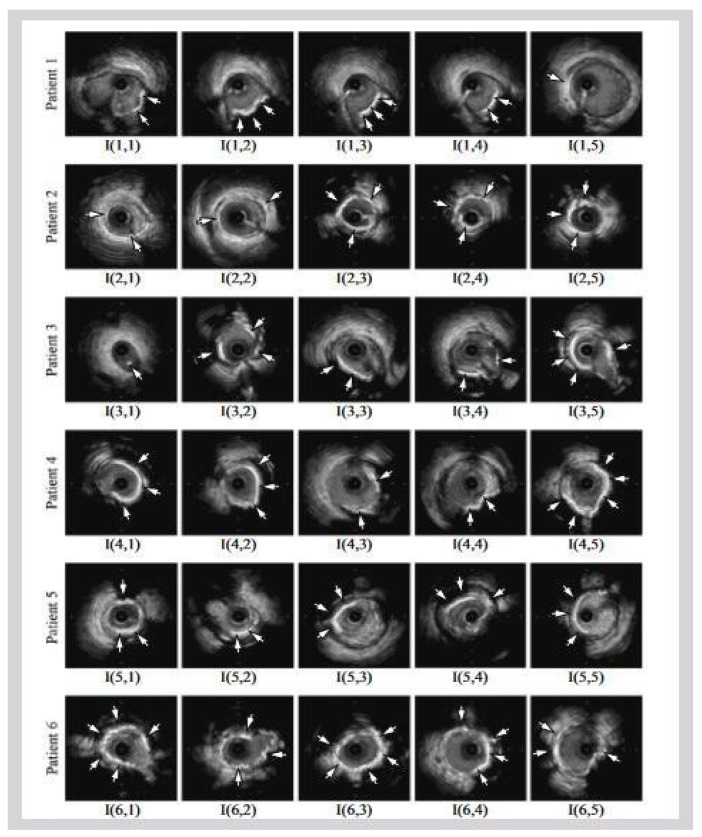
Six patients’ calcified plaques can be seen in sample frames I (1,1) through I (6,5) from the overall intravascular ultrasonography (IVUS) films [43].

Figure 1b shows a popular imaging method for the assessment and control of CVD, intravascular ultrasound (IVUS) [5,26]. In conjunction with positional data, IVUS images are segmented into interior and exterior regions as lumen and media regions, respectively. Arteries’ representation in 3D heavily depends on arterial vessel walls for various purposes such as surgical planning. The arteries’ segmentation is helpful for plaque identification in clinical practices. IVUS-guided percutaneous coronary intervention (PCI) is a more advanced and superior technique in comparison to standard angiography-guided PCI, minimizing death risks in patients [6]. IVUS segmentation for lumen and vessel cross-sectional based on 3D vessel reconstruction is precise and quick for accurate and real-time segmentation during PCI [27]. However, IVUS segmentation requires recent, accurate, and faster techniques, typically at 30 Hz and 100 Hz frame rates. To record an IVUS sequence, a catheter-borne ultrasound transducer is inserted into the coronary artery and then returned via arteries at a speed of roughly 1 mm/s [5]. Raw radio frequency (RF) information from the probe is typically not used for analysis. However, amplified and filtered gray-scale B-mode Euclidean ultrasound pictures showing the coronary cross-section provide a typical output format for downstream evaluation (see Figure 2) The arrows in Figure 2 depict a typical example of five (1–5) frames with calcified plaques. Six patients’ IVUS videos’ worth of frames were collected, and they were placed in a 6 × 5 matrix. The symbol I (1,1)-I (6,5) is used to represent this.

IVUS segmentation is one of the most challenging tasks in medical images. It consists of lumen–intima (LI) and media–adventitia (MA) border detection. This challenge is due to the presence of the artifacts, namely shadows, bifurcation, and echogenic plaques, and the fact that public expert-labeled ground-truth databases only contain a small number of captures [28]. Even though artificial intelligence (AI) has shown promising signs toward higher accuracy and learning strategy, it has been observed that these AI-based black boxes lack clinical validation and the ability to perform well in clinical settings, and they are unable to explain the outcomes [29,30,31,32,33,34,35]. The clinical validation requires that the outcome from the AI system must have a behavior leading to correct coronary artery disease risk assessment. For example, should an AI system perform accurately on a test patient who has a high risk, then the syntax score of this patient should be high [36]. Other ways to show the clinical validation include by estimating the relationships or correlations between two quantities such as computed tomography (CT) coronary artery score vs. AI outcome of the risk [37]. Such consistent behavior needs to be exhibited by AI systems. Other than the clinical validation, there are attributes such as imbalanced classes in the datasets that can introduce AI bias [38]. Such causes can lead to bias in AI modules or system designs.

The ability of UNet-based deep learning models as shown before is very powerful in the imaging domain and can handle image noise, structure, scale, size, resolution, and further, the variability in the shapes [39]. Thus, we applied that as an assumption to hypothesize that UNet-based solutions are more powerful than conventional models for wall segmentation in IVUS scans. The second component of the AI-based solutions is the ability to explain the output results due to input variations. Its explainability has been successfully applied in immunology contexts [40,41,42]. We hypothesized that once AI explainability is applied, it will help elucidate the internal design of the AI system for wall segmentation in IVUS. Therefore, we hypothesized that a similar trend could be observed in our studies, which means the IVUS model is likely to be biased. Second, with the evolution of AI, it has been observed that the fusion of the techniques leads to superior performance [18]. Thus, we hypothesized that the deep learning (DL)-based UNet AI model is likely to provide a superior performance as compared to the non-UNet (conventional) method. This review study addresses conventional and AI-based UNet methods of coronary artery wall segmentation in IVUS scans, integrating the three AI dimensions: explainable AI (XAI), risk of bias (RoB), and DL-based pruning among DL systems.

The review has the following layout. Section 2 presents the PRISMA model for study selection and the statistical distribution of the AI attributes used in the study. Section 3 shows the classification tree for the AI-based method for wall segmentation. Section 4 represents the RoB estimation in a deep-learning-based coronary artery disease system. The explainability of the AI system is represented in Section 5. The pruning approach is covered in Section 6. The critical analysis of this research is presented in Section 7. Finally, the conclusions of the review are summarized in Section 8.

## 2. Search Strategy and Statistical Distribution 

To comprehend the various CAD methodologies, the gold-standard modifications regarding such machine learning solutions, the involvement of the feature extraction methodologies, and bias in AI-based approaches, it is vital to grasp the statistical distribution of the literature. In order to choose the studies for CAD wall segmentation in IVUS scans, we adopted the PRISMA model. Consequently, this section is split into two sections: The research selection criteria are covered in Section 2.1, and the statistical distributions are covered in Section 2.2. 

### 2.1. PRISMA Model

**Figure 3:**
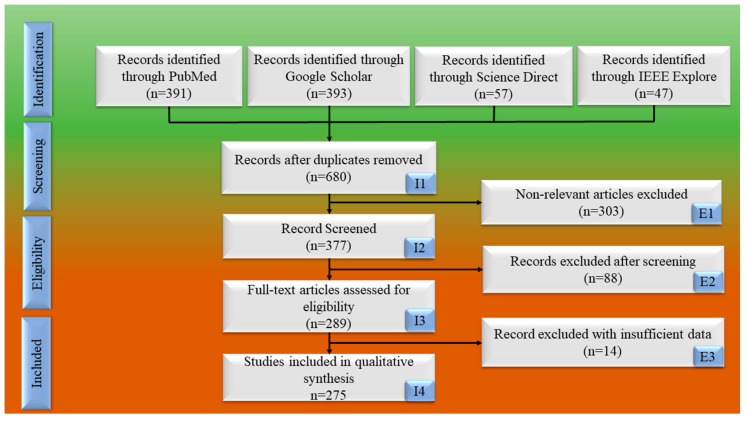
PRISMA paradigm for IVUS imaging selection of UNet-based CVD investigations.

We adopted PRISMA strategy to determine the relevant studies in the domain. The key terms exploited are deep learning (DL) and CVD. In addition, relevant terms such as “CAD risk using DL”, “CAD risk stratification in DL framework”, “CVD risk estimation using AI”, “CVD/stroke risk analysis in DL model “, “CAD/Stroke utilizing non-invasive framework”, “Bias in Deep learning/Artificial intelligence for CVD risk stratification”, “IVUS segmentation and DL”, “IVUS segmentation using UNet”, and “Modality used for wall segmentation” were used. Science Direct, IEEE Xplore, Google Scholar, and PubMed were the various search engines used. Figure 3 displays the PRISMA flow chart for a few investigations. A thorough search turned up 888 studies in all. The three exclusion requisites included (a) research that was not pertinent (I1); (b) publications that, after a search, were excluded and screened from the research (I2); and (c) records with insufficient data (I3). The exclusion criteria were applied, and 303, 88, and 14 studies were identified as meeting E1, E2, and E3 (see Figure 3). E1 implies non-relevant articles, E2 is records excluded after screening, and E3 are the records having insufficient data. From these concluding studies, significant scientific knowledge was acquired (I4), and a statistical classification was developed. The architectural style of UNet techniques as well as their traits and bias estimation were analyzed. 

### 2.2. Statistical Distribution Analysis

**Figure 4:**
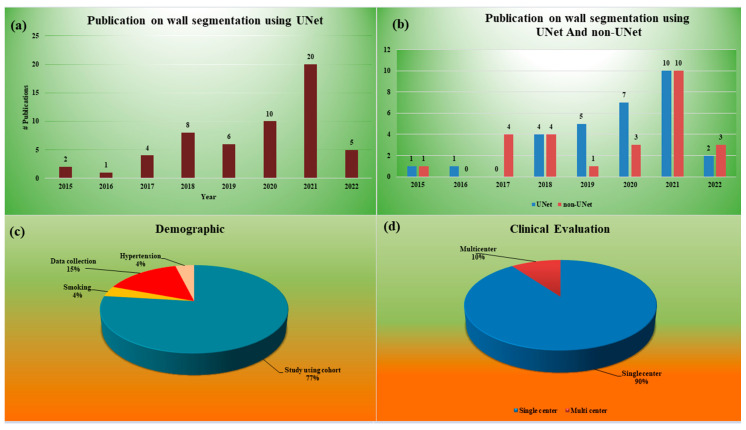
Statistical distribution: (**a**) publications per year (combined) and (**b**) publication per year (separate for UNet and non-UNet); (**c**) types of demographic; (**d**) clinical evaluation.

Since the focus of our study is on a UNet-based deep learning system for wall segmentation in IVUS scans, it is, therefore, necessary to know what has been the trend in the area of UNet-based solutions for wall segmentation in IVUS scans. This trend gives insight into the contributions of UNet-based systems for wall segmentation in IVUS scans. It also helps to elucidate the importance of UNet-based systems for IVUS applications. This is the main context for understanding the statistical distribution analysis. Figure 4a,b discuss such statistical distributions.

When considering the AI-based applications, data collection is important. The data collected for AI applications play an important role in risk stratification for coronary artery disease. These data are for humans. Therefore, where the patients come from and what kind of disease is prevented in the data are important components for the design of the AI system and its validation. Therefore, one needs to know the distribution of the demographics of the data. Figure 4c presents the distribution of the demographics of the patients. 

Another important attribute of an AI-based system is if the study used data from a single medical center or if the data were collected from multiple medical centers or institutes. Thus, it is important to know if the AI system was using data from a single center or a group of centers. Typically, the single-center data are likely to be more biased compared to that from a multiple-center study. Thus, the role of a single vs. multiple center study is exhibited in Figure 4d. 

**Figure 5:**
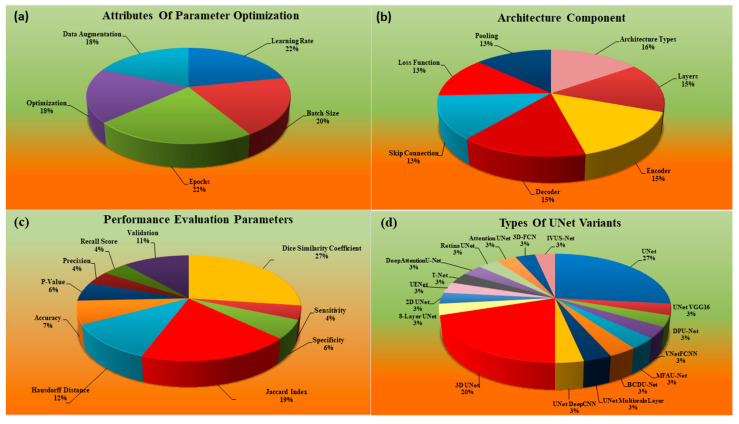
Statistical distribution: (**a**) types of attributes of parameter optimization; (**b**) architectural details; (**c**) performance evaluation; (**d**) types of UNet variants.

There are other statistical distributions that play an important role when designing AI systems. These attributes are the types of parameters used for the optimization of the AI systems, the type of the design of the AI system itself, how many studies really underwent the performance evaluation of the AI system, and finally, what kind of variation was used in the design of the UNet-based deep learning system. Thus, there is a clear need to know how the trend has been when using AI-based solutions for wall segmentation in IVUS scans. Such behavior is shown in Figure 5a–d.

The statistical distributions and analysis of the selected studies is demonstrated in Figure 4 and Figure 5. The percentage reflects the number of studies used for that parameter out of the total studies, which number 60 in this case. For example, a sensitivity of 4% is 4/100 × 60 = 2.4 or 2. This means only 2 out of 60 studies computed sensitivity. The statistical distribution in Figure 4 illustrates the following parameters: (a) combined (both UNet and non-UNet) publications over the year, (b) separate publication trend for UNet and nonUNet the over year, (c) demographical attributes used in the study, and (d) clinical evaluation. The DL-based publications showed that a lot of work was carried out in this area from 2015–2022, as shown in Figure 4a,b. Figure 4c illustrates that the percentage distribution of the number of studies that considered demographic attributes include 77% [24,44,45,46,47,48,49,50,51,52,53,54,55,56,57,58,59,60,61,62] patients, 4% [59] smoking, 15% [45,53,63,64] data collection, and 4% [58] hypertension. Figure 4d shows clinical evaluation in the DL system. Two sets were considered, namely single center 90% [24,26,28,44,46,47,48,49,50,51,52,53,54,55,56,57,59,60,61,62,64,65,66,67,68,69,70,71,72,73,74,75,76,77,78,79,80,81,82,83,84,85,86,87,88,89,90,91,92,93,94] and multicenter 10% [45,58,95,96,97,98]. The statistical distribution in Figure 5 details the parameters, namely (a) the attributes of parameter optimization, (b) the architectural design followed in the DL-based paradigm, (c) various performance metrics utilized in the CAD segmentation of IVUS scan, and (d) different variants of UNet adopted for CAD segmentation. In Figure 5a, the DL systems were also analyzed by considering the percentage distribution in parameter optimization, including 22% [24,47,48,51,53,54,55,56,58,59,60,61,62,64,67,68,69,71] learning rate, 20% [24,46,47,48,50,51,53,54,55,56,58,59,61,62,67,69,71] batch size, 22% [24,45,46,47,48,50,51,53,54,55,56,58,59,60,62,64,67,69,71] epochs, 18% [24,54,55,56,57,58,59,60,61,62,66,67,71] optimization, and 18% [45,46,49,50,51,53,54,58,59,61,62,66,67,68,71,73] augmentation.

Figure 5b displays the percentage distribution of the architecture details of the DL system comprising 16% [24,44,45,46,47,49,50,51,52,53,54,55,56,57,58,59,60,61,62,64,66,67,68,69,70,71,97] architecture used, 15% [24,44,45,46,47,49,50,51,52,53,54,55,56,57,58,59,60,61,62,64,66,67,68,71,97] layers, 15% [24,44,45,46,47,49,50,51,52,53,54,55,56,57,58,59,60,61,62,64,66,67,68,71,97] encoder, 15% [24,44,45,46,47,49,50,51,52,53,54,55,56,57,58,59,60,61,62,64,66,67,68,71,97] decoder, 13% [24,44,46,47,48,49,50,51,52,53,54,55,56,57,59,60,61,62,64,65,66,67,68,70,71] skip connection, 13% [24,44,45,47,48,49,50,51,52,53,54,55,56,57,59,61,62,64,67,68,70,71] loss function, and 13% [24,44,45,46,47,48,49,50,51,52,54,55,57,58,59,60,62,64,67,68,69,70,71,99] pooling. The DL systems were also analyzed by considering their evaluation of performance.

The Figure 5c shows the percentage contribution where the Dice similarity coefficient (DSC) was 27% [24,44,46,47,48,49,50,51,52,53,56,58,59,60,62,67,68,70], validation 11% [47,55,59], recall score 4% [24,62,67], precision 4% [24,48,62], and sensitivity 4% [24,62,67]; *p*-value was at 6% [57,58,59,62], specificity 6% [44,48,52,67], accuracy 7% [24,45,46,62,67], Hausdroff distance 11% [44,47,53,54,55,58,59], and Jaccard index 19% [44,45,46,47,54,55,56,61,62,66,67,70,71]. These are the pillars that stabilize the DL system, designed to prevent it from showing biased in machine learning models. For the best results, it is necessary to investigate these ML traits. 

The Figure 5d depicts the percentage contribution of the different variants of UNet in the DL framework, with 20% comprised by 3D UNet [24,49,51,59], 27% UNet [45,52,54,62,67,68,69,73], 3% UNetVGG16 [46], 3% dual-path UNet [55], 3% VNetFCNN [53], 3% MFAUNet [71], 3% BCD UNet [72], 3% UNet multiscale layer [47], 3% UNet DeepCNN [48], 3% eight-layer UNet [61], 3% 2D UNet [60], 3% UE-NET [64], 3% T-Net [100], 3% attention UNet [57], 3% 3D-FCN [56], and 3% IVUS-Net [66]. 

## 3. Methodology

The wall of the coronary artery consists of three layers, namely, the intima-layer (the inner-most layer), media-layer (middle layer), and the adventitia-layer (the outermost layer). These three layers are observed in a cross-sectional view of artery in the heart, as demonstrated by IVUS imaging (Figure 2). Segmentation of walls in IVUS scans has been in existence for the past two decades using computer vision techniques [9,28,101]. Several traditional image processing approaches, such as active surfaces [5], graph search [102], and active contours [5,103], have been applied to segment IVUS images. These techniques are based on both local as well as global attributes within a grayscale image [102]. Three-dimensional fast-marching method under the umbrella of level sets incorporating the texture and the grey-level contour has been used to partition the walls of the coronary artery using a dynamic initialization of external elastic membrane (EEM) borders [104,105]. These are conventional paradigms since they do not utilize any knowledge-based system for segmentation. We thus categorize them as non-AI based models. 

**Figure 6:**
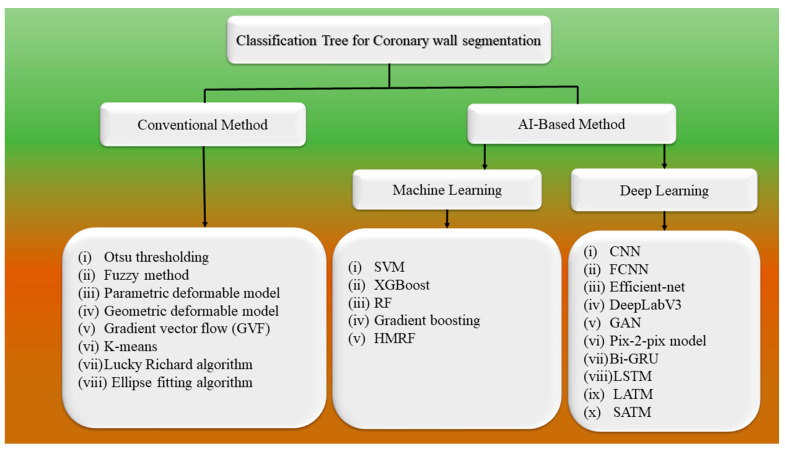
Classification tree of IVUS segmentation. SVM, support vector machine; RF, random forest; HMRF, hidden Markov random field; CNN, convolutional neural networks; FCNN, fully convolutional neural network; GAN, generative adversarial network; Bi-GRU, bidirectional gated recurrent unit; LSTM, long short-term memory; LATM, location-adaptive threshold method; SATM, scan-adaptive threshold method. Conventional method: (i) Otsu thresholding [90], (ii) Fuzzy method [87,89], (iii) Parametric deformable model [92], (iv) Geometric deformable model [92], (v) Gradient vector flow (GVF) [94], (vi) K-means [43], (vii) Lucky Richard algorithm [84], (viii) Ellipse fitting algorithm [28]. Machine Learning: (i) SVM [65,82], (ii) XGBoost [79,107,108], (iii) RF [65,82], (iv) Gradient boosting [85], (v) HMRF [43,109,110]. Deep Learning: (i) CNN [78,81,95], (ii) FCNN [87], (iii) Efficient-net [75], (iv) DeepLabV3 [80], (v) GAN [74], (vi) Pix-2-pix model [74], (vii) Bi-GRU [74], (viii) LSTM [97] (ix) LATM [111] (x) SATM [111].

There is a shift in paradigm towards AI-based models, which is the primary focus of this study [106]. To understand this better, these two frameworks are discussed in the form of classification tree, having various conventional (non-UNet) and non-conventional (UNet) methods for the segmentation of IVUS scans of arterial walls. Supplemental Material Tables S1 and S2 tabulate the conventional/non-UNet- and UNet-based deep learning works in CVD. Conventional methods include techniques such as Otsu thresholding [90], fuzzy [87,89], parametric deformable model [92], geometric deformable model [91,92], and gradient vector flow (GVF) [94]. For the segmentation of the coronary walls, various AI-based techniques, such as ML-based or DL-based, have been applied. The ML-based method includes XGBoost [79,107,108], k-means [43], hidden Markov random field (HMRF) [43,109,110], support vector machine (SVM) [65,82], random forest (RF) [65,82], fuzzy c-means (FCM) [43,89], Pix2Pix model [74], ellipse-fitting algorithm [28], Lucky–Richardson algorithm [84], and gradient boosting [85]. The DL-based method includes generative adversarial network (GAN) [74], convolutional neural network (CNN) [78,81,95], bidirectional gated recurrent unit (Bi-GRU) [74], efficient net [75], DeepLabV3 [80], location-adaptive threshold method (LATM) [111], scan-adaptive threshold method (SATM) [111], and fully convolutional neural network (FCNN) [87]. In recent years, DL has been extensively used in medical imaging analysis and achieved impressive results [73,112]. It has been used to identify the LI and MA borders in IVUS due to its advanced features such as automatic feature extraction [113,114]. A summary of the technique is shown in Figure 6. 

XGBoost is a machine learning method like other models such as support vector machine (SVM), naïve Bayes (NB), k-nearest neighbor (KNN), logistic regression (LR), random forest (RF), decision tree (DT), etc. This is a standard solution. Recently, we used XGBoost for classification protocol [107,108]. In [107], the authors presented the usage of XGBoost as a machine learning strategy for the classification of cardiovascular datasets. Similarly, in [108], the authors used XGBoost as a machine learning model for the classification of the neonatal dataset for risk stratification of premature infant deaths. These both are classic examples of the usage of XGBoost as machine learning models for classification or segmentation strategies. Very recently, there has been usage of XGBoost for ischemic stroke identification in computed tomography perfusion [115]. Another application of XGBoost has already been used in the area of diabetic retinopathy (DR) as shown by the authors [116]. Hidden Markov random field (HMRF) is another classic model that has been efficiently adopted for the segmentation and classification of different applications. Recently, our group applied HMRF for coronary artery wall segmentation as well [43,109,110]. This is a direct application of HMRF for IVUS-based coronary artery wall segmentation and is characterized as a machine-learning model.

Still, the conventional model had dominated for a long time due to UNet’s strong abilities, such as automatic feature extraction, the ability to add a transformer, and its attention-enabled solutions [39,117].

Below, the three conventional methods and three UNet-based methods are further detailed and organized as follows:

3.1. Conventional techniques

3.1.1. Fuzzy method

3.1.2. Parametric methods

3.1.3. Geometric methods

3.2. UNet-based techniques

3.2.1. MFA UNet

3.2.2. Dual-path UNet

3.2.3. Eight-layer UNet

Each of the UNet-based methods has an encoder and decoder architecture. Note that each of the methods contains a subsection.

### 3.1. Architecture for Wall Segmentation Using Conventional Methods

The conventional method for coronary artery wall segmentation includes Otsu thresholding [90], fuzzy method [87,89], parametric deformable model [92], geometric deformable model [92], and gradient vector flow (GVF) [94]. Among these methods, here, we discuss the representative work, namely the fuzzy method, parametric model, and geometric model, for wall segmentation in IVUS scans.

#### 3.1.1. Fuzzy Approach for Wall Segmentation

**Figure 7:**
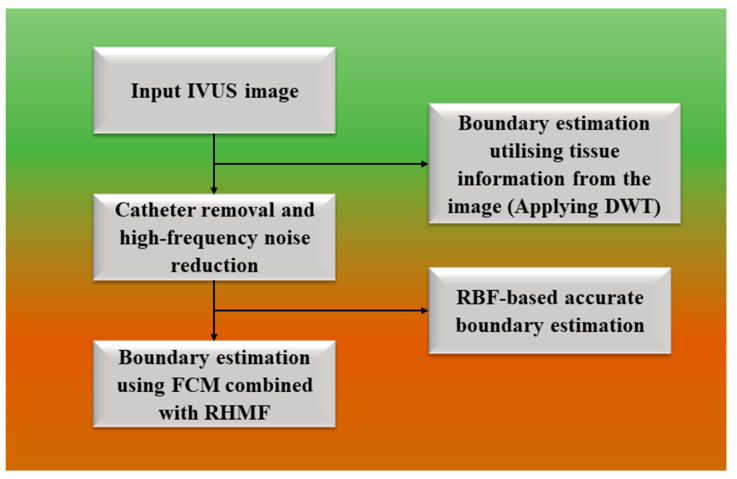
Flowchart of FCM method.

Eslamizadeh et al. [89] introduced a fuzzy approach for boundary wall segmentation for lumens in IVUS images. Figure 7 below shows the algorithm description. In order to find and remove catheters for assessing lumen boundaries during IVUS, the pre-processing stage consists of reduction in speckle noise from the image. This is accomplished using spatial filters in polar coordinates. Two integration-based fragmentation methods, such as fuzzy c-means (FCM) and robust high-order matched filter (RHMF), as well as a tissue-based boundary identification algorithm are used in the processing step to find a more precise initial border estimation. Then, in the subsequent stage, improving boundary detection while concurrently lowering fault detection is accomplished by applying approximations based on radial basis function (RBF). The fuzzy c-means (FCM) approach is also used to split images into two groups that represent the lumen’s interior and exterior. The largest detected region is identified as a lumen area as a result of discovering the region in an image using the RHMF approach, which is an algorithm to detect the object in an image. In order to obtain the final boundary, RBF generates an estimated border. A particular boundary is then found using this technique after discrete wavelet transformation (DWT) is used simultaneously to detect boundaries in images. The recommended limit of the radiologist is contrasted with the ultimate limit, and this provides an accuracy of 86.06%, showing better performance as compared to the other method. The main problem with this method is the lack of accurate boundaries. It is to be emphasized that the pre-processing processes have a major impact on how IVUS images are processed. Therefore, it was highly suggested that more advanced algorithms be created for various parts of the image.

**Figure 8:**
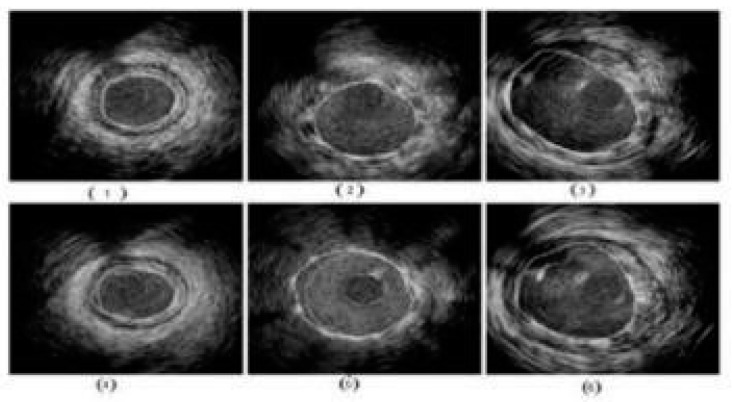
Result of FCM method [89].

The result of this FCM method is that, when compared to other methods, the median filter performed best during the pre-processing phase in reducing noise from IVUS images. This method provides an accuracy of 86.02%. The results of the detected boundary for six images using the FCM technique are depicted in Figure 8.

#### 3.1.2. Parametric Models

In this study [15,118,119], the so-called stop-and-go snake is a new geodesic snake formulation that is used to locate the calcification and soft plaque regions in atherosclerotic plaques. By using probability maps to separate regularity and convergence, this snake can better manage the function of curvature. The applied force requirement is divided into an attracting and a repulsive vector field to ensure convergence. In a conventional pattern recognition pipeline, researchers applied this new snake: The images were first processed for the extraction of texture features such as co-occurrence matrices, Gabor filters, and local binary patterns. The second step included calcium, soft and fibrous plaque treatment, and classification using AdaBoost. A probability map for the stop-and-go snake was created using the confidence rate map that was obtained using the normalized version of the likelihood map $\overset{˘}{M}$ to be employed in the stop-and-go snake.

(1) $$\frac{\delta Г}{\delta t}=\propto K\overset{˘}{M}\cdot n+\beta \langle \nabla \left(1-\overset{˘}{M}\;\right),n\rangle \cdot \;n+V_{0\;\;}\left(1-\overset{˘}{M}\right)\cdot n$$

where Г represents the snake, t represents the snake evolution at time t, K is the curvature of Г, the curve’s smoothness is controlled by weighting curvature’s function ∝ and *β*, n is its inward unit normal, < and > stand for the scalar product of two vectors, $V_{0\;}$ represents velocity. 

The choice of soft plaque is ineffective. However, calcium detection is satisfactory. Also, it is not easy to estimate the snake outcomes statistically. The results of this approach show the likelihood map for soft plaque in this example was created using the categorization confidence rates between fibrous plaque and calcium against soft plaque. 

**Figure 9:**
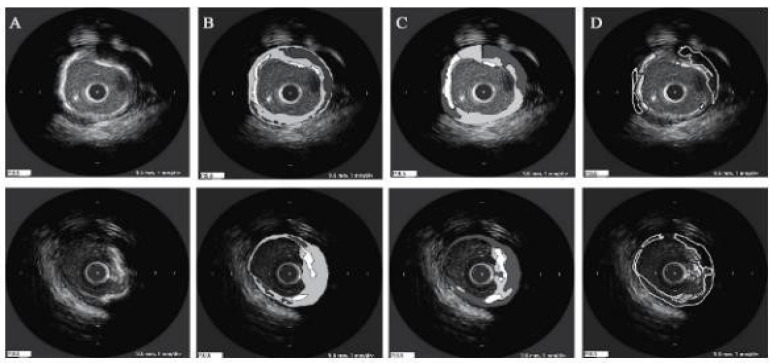
Results of classification and snake for two IVUS images showing soft plaque and calcium. Original photos are in column (**A**). (**B**) Pictures that the cardiologist segmented. (**C**) A classification map of AdaBoost. (**D**) The stop-and-go snake outcome. White lines indicate soft plaque, whereas black ones indicate calcification in column D’s legend [118].

Before using the diagram as a likelihood map, only the rates that are below a pre-defined threshold were taken into analysis, and then, the diagram was reversed. In this study, numerous pattern recognition tools were taken into consideration to achieve autonomous plaque tissue segmentation. Different textural features were retrieved, and the stop-and-go snake and the AdaBoost classifier both produced promising calcium segmentation results. Figure 9 illustrates the IVUS images showing the presence of calcium and soft plaque in the arteries.

#### 3.1.3. Geometric Approach 

By linearly projecting the inner (lumen) and outer (media) contour spaces onto a pair of low-dimensional previous form spaces at each border, this method [93] separates the lumen and media layers in arterial walls. The algorithm operates on the rectangular (without scan transformation) IVUS image domain. By adjusting the template (average) shape of the previous lumen contour region in accordance with the intensity average above the average region, a lumen layout is initially created. The occurrence of brightness within and outside the lumen $P_{in}$ and $P_{out}$, derived from a Parzen frame that is calculated by a distribution of brightness, is used to develop the lumen contour via a Euler–Lagrange equation:

(2) $$\frac{\partial \alpha_{i}^{l}}{\partial t}=\;{ꭍ}_{c}-log\left[\frac{P_{in}\;(I\left(x\right))}{P_{out}(I\left(x\right))}\right]\;U_{i}^{l}dx$$

where $U_{i}^{l}\;\;$is the eigenshape corresponding to the ith lumen structure weight $\alpha_{i}^{l}\;$, *l* represents the lumen data, and $I\left(x\right)$ is the intensity image. 

The highest smoothed gradients at regular intervals of the rectangular picture are used to create the initial shape of a media contour, which is then developed at a rate proportional to the gradient difference between two aligned windows both above and beneath the contour ∇G.

(3) $$\frac{\partial \alpha_{i}^{a}}{\partial t}=\;{ꭍ}_{c}\nabla G\left(x\right)U_{i}^{a}dx$$

where $U_{i}^{a}$ is the eigenshape corresponding to the ith media contour weight $\alpha_{i}^{a}$, *a* represents the m-a data, and ∇G represents the smoothed-oriented edge gradient.

**Figure 10:**
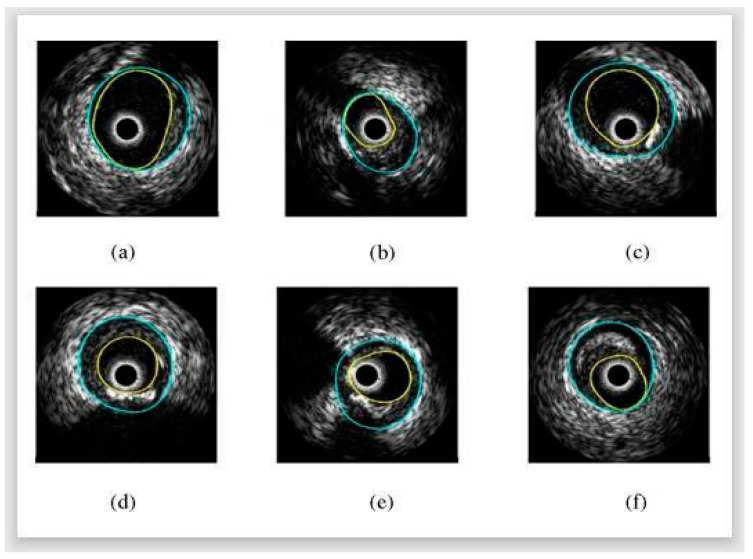
Examples of LI and MA borders with the following feature extraction. (**a,b**) opening in the branches, (**c**) calcification of the branch opening, (**d**) black shadow along with heavy calcification, (**e**) two types of calcification, (**f**) calcified bright plaque region without black shadow [93].

Before segmenting each IVUS pullback frame, calcifications and openings caused by lateral branches are detected as features to be employed in separating the borders of the lumen and media. This method exhibited slightly lower performance in the case of bifurcations. The result of this approach is that, in the “resampled” rectangular domain, the authors provided a statistical shape model-based method for separating artery walls as apparent in IVUS images. They limited the lumen and m-a contours to a smooth, closed geometry, which enhanced the segmentation quality without affecting any adaptability for a regularized term. They used a nonparametric intensity model based on an image probability density energy to segment the lumen contour as opposed to the point-wise observations of earlier techniques. Using edge information, the m-a was divided into sections. They developed an aligned, smooth gradient that eliminates the noise present in IVUS images. Additionally, they created a technique that makes use of anatomical features to find calcifications and branch openings. This segmentation method is substantially improved by incorporating the feature information into the m-a contour extraction. Figure 10 shows the result of this method. 

### 3.2. Architectural Design for 2D Wall Segmentation Using UNet-Based DL System

**Figure 11:**
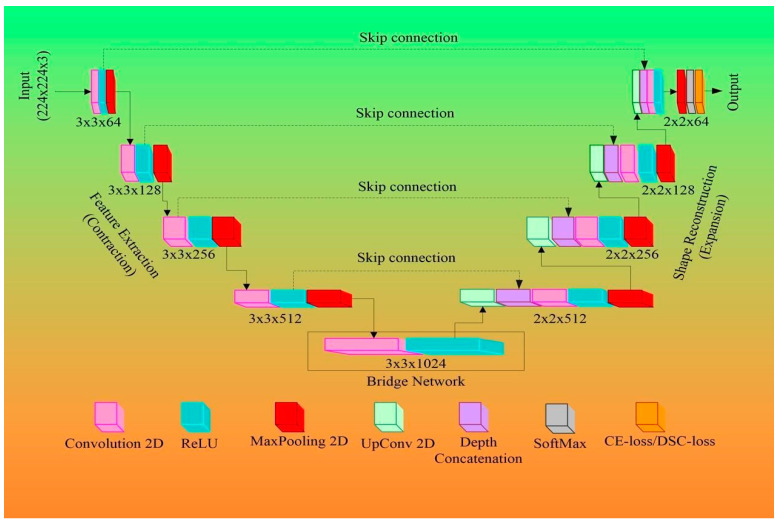
The encoder, bridge network, decoder, and skip connection are displayed in the basic UNet design.

Figure 11 describes the DL-based UNet architecture proposed by Ronneberger et al. [73]. Four encoders and four decoders are typically arranged in a “U” form along each side. The specific size of the mini-batches of gray-scale images along with masked binary ground GT is supplied as input to UNet. The size of the mini-batch is dependent on computational and hardware specifications of the system used for training the network. In [73], the authors used a mini-batch size of 10 images. The encoder and decoder modules are explained below.

The Encoder Block

The encoder blocks, the bottom levels of the UNet architecture, are used to extract the features of the image. The feature extraction procedure is carried out via convolution and ReLU procedures. The highest features in each filter zone are chosen in the “max-pooling” block, the last stage of each encoder, before down-sampling the image even more. As a result, the convolution (pink), ReLU (aqua), and max-pooling (red) operations are applied to each of the UNet’s layers on the encoder side a second time (see Figure 11). Figure 11 depicts 64 filters, which increase in size by a factor of two at each subsequent level for a total of 128, 256, and 512 filters, respectively. Figure 11 represents the numbers 3 × 3 × 64, 3 × 3 × 128... 3 × 3 × 1024, where 3 × 3 is the filter size, and 64...1024 is the number of filters.

The Decoder Block

The decoder stages are shown in Figure 11 on the right. The encoder block has been turned around. The original proportions of the training image must be retrieved. The decoder module’s filters, on the other hand, use the numbers 512, 256, 128, and 64 to divide each level in half. These filters are used to resize the image to its original specifications. The decoder generates the image with improved features that are easy to extract. The decoder stage has a number of layers, such as up-convolution-2D (light green), depth-concatenation (light purple), 2D convolution (pink), and ReLU (aqua) (Figure 11). These filters are followed by the softmax layer, which converts the output to a binary image with a foreground (white) and background (black).

#### 3.2.1. MFAUNet

This architecture was proposed by Xia et al. [71]. In this UNet variant, the multi-scale skip connections is altered by addition of feature aggregation module (FAM) block. The FAM uses a bi-directional convolutional long short-term memory (BConvLSTM) unit [71] to extract context information from a spatial–temporal perspective. Additionally, with the use of multi-scale inputs and thorough supervision, each encoding and decoding phase is provided significant access to the original source and result. The “multi-scale feature aggregated UNet (MFAUNet)” is the name given to this network.

Architecture of MFAUNet
Encoding and Decoding Path

**Figure 12:**
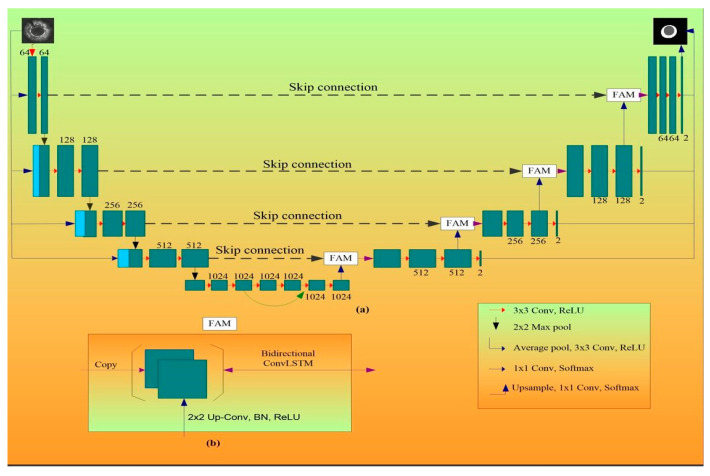
(**a**) The design of the MFA-UNet. The width and height of each box represents the number of feature channel and the spatial extent of the feature map. (**b**) The feature aggregation module (FAM). The number of feature channels and the spatial extent of the feature map are represented by the width and height of each box, respectively. (**b**) The feature aggregation module (FAM).

Figure 12a shows the full architecture of the MFA-UNet. The input is processed through the encoding path’s five phases to identify both fine and coarse features. Four stages make up the decoding path, which restores the spatial resolution to produce the final prediction. One block made up of two successive convolutions is included in each of the top four encoding levels. In the bottom layer of the contracting path, there are three blocks packed tightly together. Features from the second block’s learning are mixed with features from the first block’s learning and repeated before being transferred to the third block. The network can learn different characteristics, backpropagate gradients effectively, and allow for better information flow thanks to the network’s dense connections [13]. Each encoding layer can directly extract characteristics from the source when the multi-resolution image pyramid is input. Direct access to the source makes it possible to represent intermediate features more effectively because successive convolutions and max-pooling contractions have the potential to lose fine object information [14]. In order to increase the collective learning process, we must also impose intense oversight over the decoder. The deep supervised model maintains semantic discrimination in the hierarchical decoding layers at all stages [6].

Feature Aggregation Module.

The high-resolution local information is contained in the features copied from the appropriate encoding layer in the skip connection, whereas the global semantic information is contained in the features retrieved from the prior up-convolutional layer [12]. Concatenating features at the feature dimension is all that the conventional UNet does. Concatenation restores information that was lost during cascaded encoding procedures. MFAUNet adds a non-linear FAM adopted from the BCDUNet [15] for better feature fusion and information preservation, as seen in Figure 12b. Recurrent neural networks (RNNs) of the type ConvLSTM can remember previously learned information, handle complex object distributions, and capture spatiotemporal relationships of sequential data [16]. By utilizing two ConvLSTMs, both forward and backward input are fully taken into account by the BConvLSTM, increasing the accuracy of predictions [15]. The BConvLSTM can be used to combine the feature sequence from a spatiotemporal perspective and provide the FAM with the picture context information. 

The advantage of this network is that in order to achieve sufficient learning from a limited number of detailed IVUS photos during the training phase, the MFA-UNet integrates a FAM module, multiscale inputs, and deep supervision into the UNet model. This allows for the simultaneous extraction of the MAB and LIB in IVUS images. In this study, the MFAUNet is optimized using the focal Tversky loss.

#### 3.2.2. Dual-Path UNet

Yang et al. [55] introduced dual-path UNet architecture for the delineation of arterial walls in the IVUS scans. This design architecture is based on UNet and consists of two main parts [73]. One part is the encoder network that generates a low-resolution deep feature map after downsampling the input. The other part is the recovery part from a decoder network that restores the deep features to its original resolution and size.

The decoder network has five decoding blocks compared to the encoder network’s six encoding blocks. Each block in the network, beginning with the second block, receives the feature map from the layer before it. Each decoder layer also includes a separate skip link, which can be utilized to transmit information from the encoder networks. The skip links between the encoder and decoder networks offer extra information that can be used to enhance feature map size.

**Figure 13:**
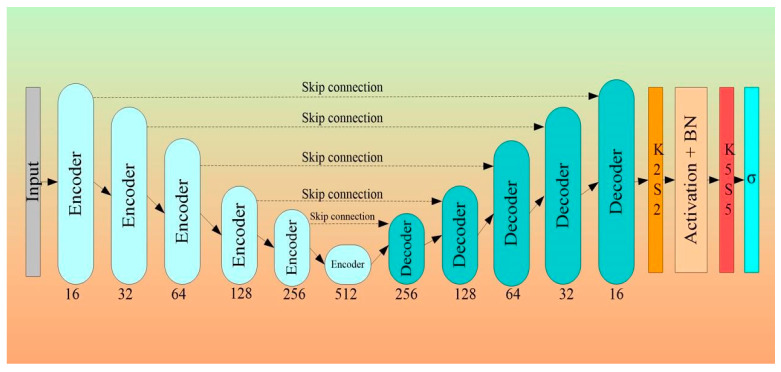
The DPUNet architecture.

Skip connections in the network preserve spatial linkages among pixels in IVUS imaging by integrating the corresponding encoder and decoder layers. Skip connections can also accelerate training and avoid vanishing grading limitations of the deep network [120,121]. Figure 13 represents the dual-path UNet architecture. It can be observed that this network is symmetrical in construction.

**Figure 14:**
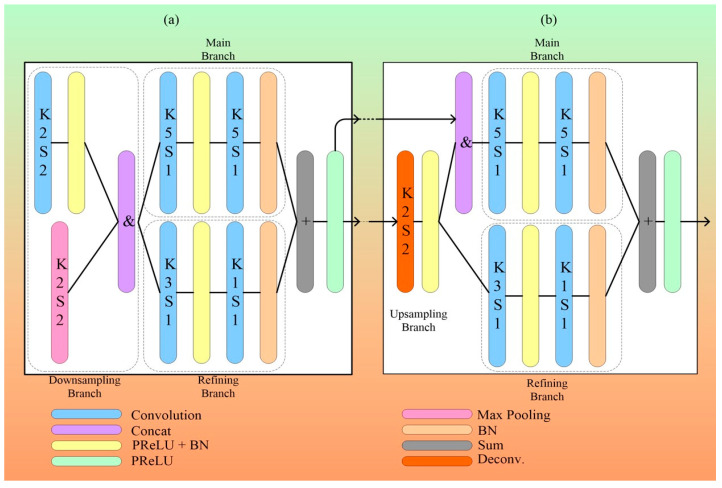
(**a**) The main branch and the refining branch are followed by an encoding block with a downsampling branch; (**b**) a typical decoding block that takes feature maps from the skip connection as well as the preceding block. The following acronyms are used in the figure: K2S2, kernel size 2 and stride size 2; BN, batch normalization.

Figure 14a illustrates encoding block with downsampling and refining branch. The downsampling branch consists of a 2 × 2 max-pooling layer and 3 × 3 convolutional layer. Max pooling is suited best for the IVUS images that are of low resolution and blurry. They save data from the most active neuron in a small-sized kernel by discarding the irrelevant information. To reduce the impact of information loss due to the pooling layer, a stride of 2 is applied to minimize image spatial resolution. The depth layer aggregates the input from these two branches [122,123]. 

The main branch and refining branch receive the integrated image feature map generated by the downsampling branch. The main branch consists of a convolution layer, activation, and batch normalization, as represented by [73,124]. Utilizing tiny kernel sizes for feature map refinement is a recent trend [125,126], and the literature frequently makes use of the idea of networks in networks [122]. To create a similar but more refined feature map, we therefore propose a refining branch that contains one convolution operation with a 3-by-3 kernel size succeeded by a convolution operation with a 1-by-1 kernel size. Since a 1-by-1 convolution only affects one pixel and is therefore unaffected by its neighbors, it can be used to trim or improve a feature map, although, in terms of total depth, this idea is superior to the global average pooling with more learning potential [63]. Additionally, since it is typically desirable to be able to capture features at different sizes, we configured convolutional layers with a kernel size of 5 in the main branch as compared to 3 and 1 in the refining branch. The following block and its associated decoding block receive the whole of the outputs from the main and refining branches. Deep networks are challenging to train because of the gradient vanishing problem, which is a serious challenge. In addition to offering a suitable local architecture, the multi-branch and local networks-in-network architecture also strengthen the gradient flow to quicken training.

A slightly different structure is required for decoding blocks, as depicted in Figure 14b. The feature map is given to each decoding block from both its preceding layer and its corresponding encoding layer. A 2 × 2 transposed convolution is used to upsample only the feature map obtained from the preceding layer, which is then integrated with the feature map from the matching coding block. Keep in mind that the main branch will be the only one to handle this concatenated feature map, while the refining branch just processes the unsampled feature map. The parametric rectified linear unit (PReLU) [121] is the activation employed in the DPUNet.

PReLU(*x*) = max (0,*x*) − *α*max (0, −*x*) (4)

ReLU only conveys gradients when the neuron is active, whereas PReLU [97] permits a portion of the gradients to pass through the neuron when it is not engaged. In several benchmarks, PReLU improves ReLU and also has a more consistent performance, as shown in [127,128].

The next step, which has been experimentally shown to improve performance, is to modify the output feature diagram from the last decoding block using a 5 × 5 convolution layer after it has been upsampled by a 2 × 2 transposed convolution layer having a 2-stride size. The resulting final outputs are identical in size to the pictures from the training dataset because of the additional 2-by-2 transposed convolution layer. A sigmoid function is used as the activation function after the final convolutional layer to produce the final binary masks.

It is also important to note that the skip connections between the respective encoding and decoding layers provide additional gradient flow to the existing design as well as context information for the decoder layer.

The result of using this network for the segmentation of arterial walls in IVUS scans, i.e., DPUNet, a fully convolutional deep network, is the ability to generalize even when there are few training samples. For the 40 MHz and 20 MHz datasets, respectively, DPUNet improved JM accuracy by more than 4% to 5%. By contrasting it with two other general-purpose feature extraction architectures, SegNet and UNet, that were trained over an identical number of images for the same period without performing any augmentation, they were able to assess the proposed DPU–generalization network’s capacity.

#### 3.2.3. Eight-Layer UNet

In this variation of UNet, eight layers are used instead of four layers. Apart from this, 3 × 3 convolution is used in place of a max-pooling operation.

Network architectures of eight-layer UNet

The most widely used convolutional neural network design for biomedical image segmentation is the UNet, which is one type of network that is entirely convolutional [129]. It has encoder and decoder parts that predict the segmentation results at the pixel level as opposed to classifying pictures at the image level. The encoder component extracts higher-level characteristics and is utilized for downsampling.

The output from the encoder portion is up-sampled by the decoder portion, which concatenates the extracted features of the relevant layer using a skip connection. The gradient diffusion issue related to deep layers is addressed by the skip connection. SoftMax activates the final decoder layer to generate the class binary image and recover the segment’s accurate predictions.

**Figure 15:**
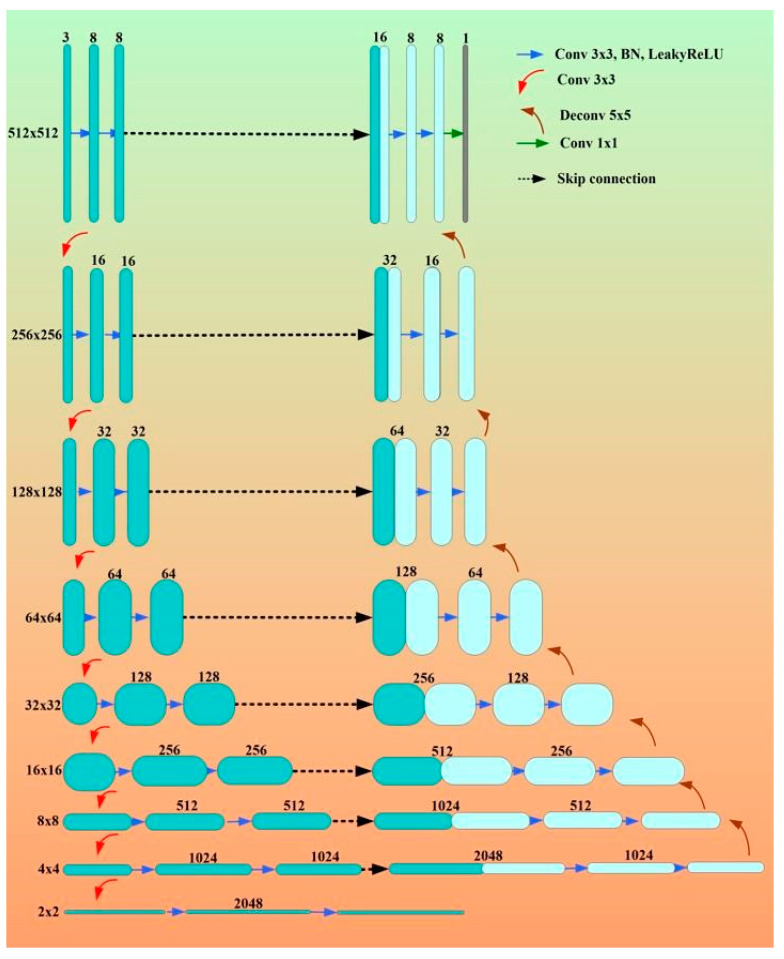
The UNet architecture with eight layers.

The nine blocks that make up the encoder component each contain two repetitions of the 3 × 3 convolution, batch normalization, and leaky ReLU activation. Feature maps are reduced by half by the downsampling procedure of 3 × 3 convolution with stride 2 × 2. To enable deeper abstract information, the eighth block is 2 × 2. For the decoder section to restore the image dimension, there are eight blocks. Every operation of upsampling includes a 5 × 5 deconvolution with stride 2. The matching feature maps are concatenated using the skip connection. The probability map of mask class prediction by SoftMax activation is produced by the final convolution. The entire architectural structure is shown in Figure 15. The random activation technique is used for parameter initialization across all model levels. This UNet did not offer any significant structural innovations over other UNet variants. The authors switched to an eight-layer network in place of the previous four-layer network so that they could extract more in-depth image information. The actual results confirmed the validity of this simple deepening design.

The result of this study shows an eight-layer UNet with meshgrid-flip-rotate data augmentation, which is specifically suitable for the challenging EEM-CSA segmentation of the coronary IVUS lumen. The outcomes of the experiment demonstrate its higher segmentation accuracy and effectiveness. Also, it provides a solid foundation for 3D-IVUS reconstruction when combined with X-ray projections, enabling fluid and dynamic research on plaques and vascular walls of coronary arteries. This is because image-based gating may be used to gate images.

## 4. Characteristics of UNet and Conventional DL Systems for CAD

### 4.1. A Special Note on Limitations of Conventional Models and Benefits of AI-Based Solutions

The conventional models adopted in image processing have existed for the last 50 years [130,131,132,133,134]. These methods were considered as generation I and II, where the methods were considered as local in nature and never used the cohort’s knowledge for the benefit for prediction on the test datasets. These methods had some inherent drawbacks, such as inability to provide an automated solution towards segmentation of the organs in complex medical images [130,135,136]. These methods were local in nature, and the noise would overwhelming and distract the computer vision algorithms [137]. Thus, the system was ad hoc in nature and could not be automated for every new incoming test image [138]. Due to these inherent challenges, the performance in these systems dropped considerately and affected the accuracy, sensitivity, specificity, Mathew coefficient, recall, precision, area under the curve, and *p*-value significance. Further, the statistical tests for evaluating the reliability and stability also did not perform well, which included the *t*-test, paired *t*-test, Bonferroni tests, Freedman test, Wilcoxon test, Poisson test, etc. [139,140,141]. The effect of such challenges lacked explainability and interpretations [41,142]. As a result, time and again, these computer vision methods started losing interest, and over time, inventions based on knowledge derived by the cohorts started to take shape.

With the invention of fundamental neural networks [143], these fundamental drawbacks started to disappear. The rapid rise of these methods has nearly dominated the field of image processing, which were then characterized into machine learning and deep learning approaches [144]. The most powerful paradigm was the addition of addition of intermediate layers between the input and output layers [145]. We could not only add a layer between these input and output layers, but we could add ones large in number and shape to these networks for superior feature extraction followed by classification or risk stratification [39]. These deep layers are a special case of machine learning, where the features extracted were limited and ad hoc and not like those of the deep layers, where the features extracted were stronger compared to machine learning model features [146]. Thus, the AI-based solution was characterized with superior performance compared to conventional models [147]. There have been over 1000 articles discussing the drawbacks of conventional methods over AI-based solutions, and it is nearly impossible to discuss each method, but the key challenges are thoroughly discussed above. We hope the reader will appreciate the depth of coverage of the author’s judgement for expressing the challenges in conventional models compared to more modern methods such as UNet-based solutions, which are deep in nature [41,148,149]. In summary, deep learning solutions offer the following benefits over the conventional models: automated feature extraction, the power of the integration of knowledge from cohorts for better segmentation and classification solutions, the ability to adjust the depth of layers, the ability to parallelize these neural networks to improve the performance and optimize these deep layers, and the ability to reduce the noise present in the images using dropout layers.

### 4.2. A Special Note on Quality Control for AI Systems 

The size of the cohort, the balancing of the class in the cohort, missing values in the cohort, scaled values of the risk factors, normalization of the factors if any, and augmentation of the raw datasets are all factors that are part of the quality control system during AI design. If the quality control is not conducted in a proper way, then the AI system may lack generalization. In other words, the training system will not be generalized. The cohort size plays a major role. If the cohort size is small, it can also cause overfitting. Thus, dropout layers help in improving the generalization. To further improve the generalization requires hyper-parameter tuning [145,150].

Table 1 tabulates the general characteristics of the DL system, described by using 26 attributes categorized into 5 clusters, namely demographic (rows A1–A3), architectural details of the deep learning model (rows A4–A10), performance evaluation (rows A11–A20), parameter optimization (rows A21–A25), and clinical evaluation (row A26). The cohort size used in different studies was very limited. The demographic factors considered by most of the studies were cohort size, smoking, and hypertension. 

The architectural details included in the AI-based system describe whether the given architecture is a conventional architecture or UNet architecture. The performance evaluation parameters used were DSC, sensitivity, specificity, Jaccard index, Hausdorff distance, *p*-value, accuracy, precision, and recall score. The parameter optimization in the DL system included learning rate, batch size, epochs, optimization, and data augmentation. The clinical evaluation considered single-center or multi-center data.

Standardized data augmentation was conducted on these images [40,148,151,152]. Data augmentation plays a crucial role in improving the generalization of machine learning models, including those used for coronary artery wall segmentation in intravascular ultrasound (IVUS) scans. These techniques help increase the diversity of the training data, making the model more robust to variations in the input data. Some specific data augmentation techniques commonly used in coronary artery wall segmentation for IVUS scans are as follows: (1) rotation from −5^0^ to 10^0^, (2) random flipping, (3) rotation to 2700, and (4) skewing [151].

## 5. Risk of Bias in Deep-Learning-Based Technologies for Coronary Artery Disease 

Due to the difference in the strength of the attributes (strong or weak), the AI system introduces a glitch, so-called bias. When AI algorithms are designed for solving wall segmentation in IVUS scans, they only cover the engineering component along with some part of performance evaluation. There are no elaborate protocols for clinical validation or engineering validation. The algorithms are purely focused on raising the accuracy of wall segmentation. There is no consideration of the clinical delivery of AI solutions. There are no inter- and intra-observer variability analyses. The system design lacks solutions for issues related to (i) handling large datasets (big data) and (ii) the reduction of the training model size (so-called pruning). Last but not the least, these AI systems are not generalized either, which means they are not tested on cohorts that are not part of the training cohorts. Such analysis is called “unseen analysis”, where the training is conducted on cohort A (data taken from hospital A) and tested on cohort B (data taken from hospital B). Such cross-validation schemes help improve the robustness of the AI system. Due to the above reasons, AI designs are not ideally suited for clinical applications. The AI systems are thus considered to be biased. These are the key motivations for conducting AI bias analysis [29,30,31,32,33,34,35,106].

Engineering Validation: Even though artificial intelligence (AI) has shown promising signs toward higher accuracy and learning strategies, it can be observed that these AI-based black boxes lack clinical validation and the ability to perform well in clinical settings, and they are unable to explain the outcomes [29,30,31,32,33,34,35]. The clinical validation requires that the outcome from the AI system must have a behavior leading to correct coronary artery disease risk assessment. For example, should an AI system perform accurately on a test patient, then the syntax score of this patient can be considered high [36]. Other ways to show the clinical validation is by estimating the relationships or correlations between two quantities such as coronary artery score vs. AI outcome of the risk [37]. Such consistent behavior needs to be exhibited by AI systems. Other than the clinical validation, there are attributes such as imbalanced classes in the datasets that can introduce AI bias [38]. Such causes can lead to bias in AI modules or system designs.

Clinical Delivery: By clinical delivery, we mean the evaluation of the AI design architecture in clinical settings. In other words, we evaluate the architecture on new test patients in clinical evaluation such as hospitals [33,140,153,154]. 

Inter/Intra-observer variability: Inter-observer variability is computed when different observers are used to evaluate the AI-based architecture design. The evaluation is based on the gold standard supplied by the expert observer. Different observers may have different judgements on the risk of the wall plaque in the IVUS scans. Thus, the output AI performance, once evaluated, can have variations between the results if different observers are considered. In intra-observer variability, the same observer evaluates the AI system at different times. Thus, under different sets of conditions, the same observer can give slightly different results, leading to intra-observer variability. The influencing factors include change in fatigue, lightening condition, and software upgrades [155,156,157,158,159].

In order to segment the artery wall in an IVUS scan, which is still in its early stages, particularly in the CAD area, DL approaches are increasingly adopting UNet-based techniques instead of traditional ones. Because of their automatic extraction-of-features paradigm, UNet-based systems perform better than conventional-based techniques, but RoB is still a problem. For bias estimation, two techniques—the ranking method and the region-based map—were applied, as discussed in Section 5.1 and Section 5.2. 

### 5.1. Risk of Bias via Ranking Method 

**Figure 16:**
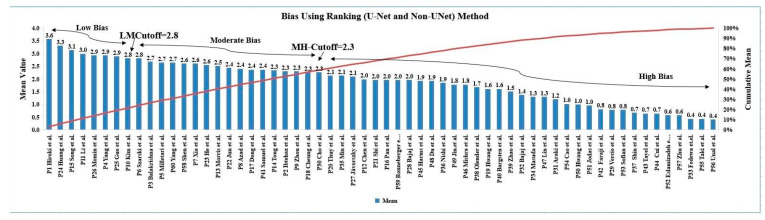
The Cumulative plot of conventional DL and UNet-based CAD studies.

The ranking approach, which is based on the means and cumulative means of the studies, was used to estimate the RoB for the DL-based systems (see Appendix A Table A1 and Table A2). These were determined by utilizing a grading method to score the respective properties. A total of 29 variables, including those related to demographics, architectural details, performance evaluation, parameter optimization, and clinical evaluation, were used to evaluate the DL-based systems. A cumulative distribution plot was computed (red line) creating MH-Cutoff = 2.3. Based on the two cut-offs—low-moderate (LM) and moderate-high (MH)—all sixty DL-based investigations were divided into three categories: low bias (7 studies), moderate bias (18 studies), and high bias (35 studies). As seen in Figure 16, the LM cut-off value was 2.8 and the MH cut-off values 2.3. High bias was caused by a lack of information regarding in family histories, smoking, ethnicity, and clinical settings as well as by precision and recall issues.

### 5.2. Radial Bias Map Method

**Figure 17:**
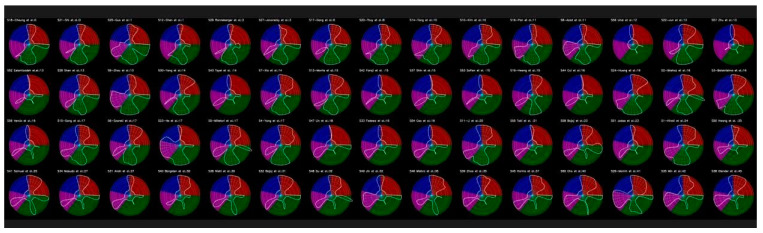
Radial Bias Map Method.

To analyze the bias in these DL systems, it was necessary to ascertain the importance of the AI characteristics (A1 to A34) that were utilized to create the UNet paradigm of wall segmentation in the IVUS scan. Different clusters, including those for demographics, architectural characteristics, performance assessment, and parameter optimization, were employed to describe these AI properties. In these four clusters, the distribution of AI traits is 6, 7, 11, and 10, respectively. The “spokes and wheel model”, which was visualized, was used to measure the strength of these AI traits in 360 degrees. Each spoke in this model represents the weights times the spoke radius as a product. The weights of the AI traits are represented in the weight matrix, which was built by knowledgeable AI experts based on their judgment. Every study contains 34 attributes in total, one for every 10.5 (360/34) degrees. The smooth curve was created by fitting the Bezier spline curve across each spoke’s termination. In a 4 × 15 grid for 60 DL experiments, the four sectors of the curve in the radial bias map are like the wings of a butterfly (which represent four clusters), as seen in Figure 17. The order of these investigations is from low to high bias, with each study’s bias shown in the map’s corner. (The bias map’s name is given as “Sn-Name: BiasValue”, for instance, “S9Zhou:13”, where 9 is the study’s number, and Zhou is its first author). The order determined by these weights is displayed in the ranking table (see Appendix A).

### 5.3. Radial Bias Area Method

**Figure 18:**
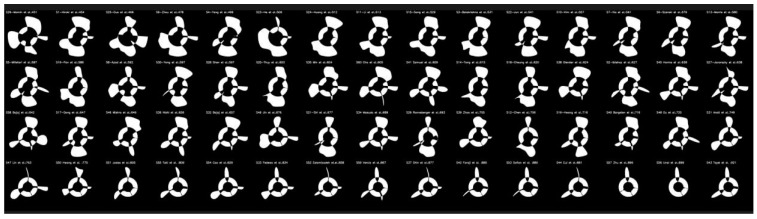
Radial bias area for DL-based studies.

To estimate the regional bias area (RBA), the difference between the regions with the best and worst DL attribute performance was used [153]. Figure 18 shows the RBA by increasing order of bias area for each of the 60 DL-based IVUS wall segmentation tests (white region). Each study’s bias is shown as “Sn-Name: Bias Value”, for instance, “S18-Cheung et al.: 620”, where “18” stands for the study’s number, “Cheung” for the study’s first author, and “620” for the bias’s normalized value. The amount of area that correlates to bias increases with the height of the white shaded region. In Appendix A, the ranking table is displayed. 

### 5.4. Comparative study of Three Bias Strategies Based on Venn Diagram

**Figure 19:**
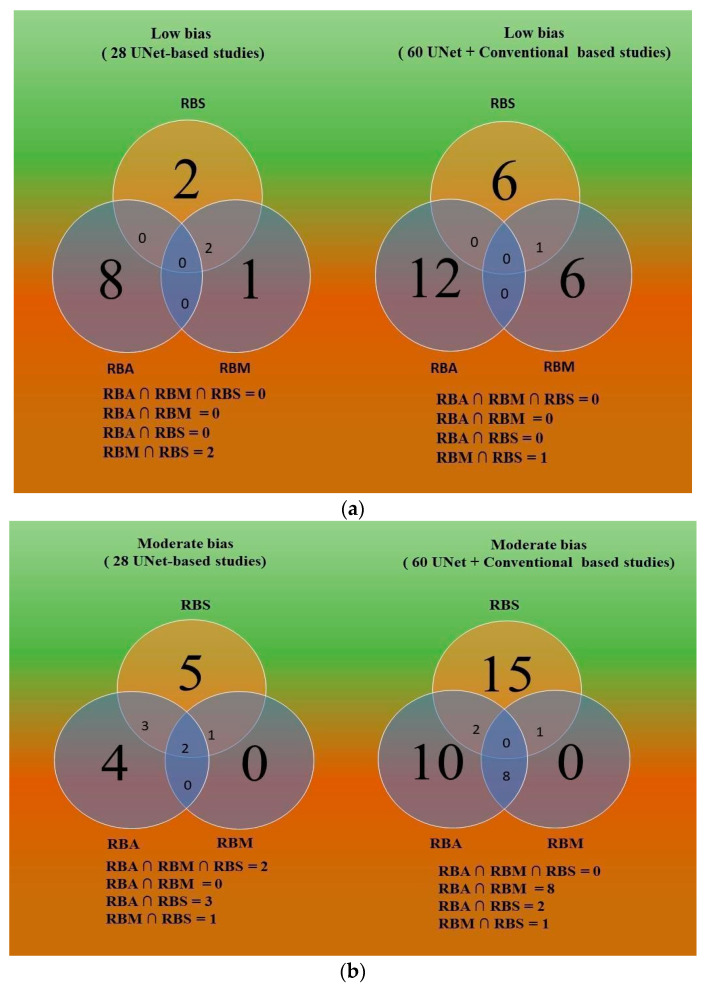

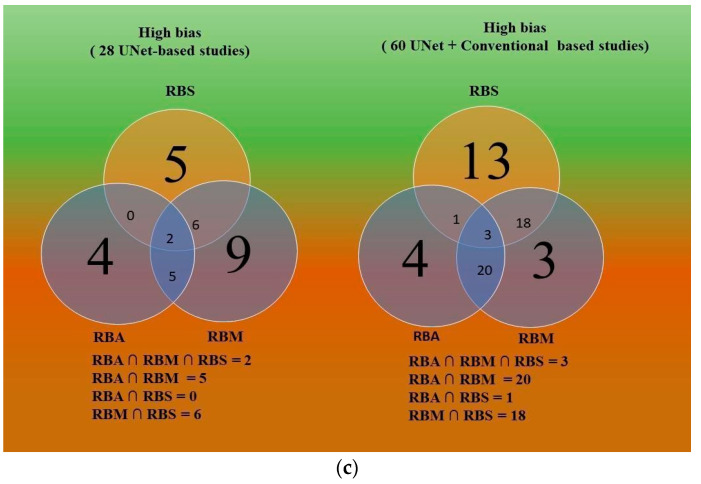
(**a**) Venn diagram showing low bias in 28 (UNet) vs. 60 (UNet + conventional) studies. (**b**) Venn diagram showing moderate bias in 28 (UNet) vs. 60 (UNet + conventional) studies. (**c**) Venn diagram showing high bias in 28 (UNet) vs. 60 (UNet + conventional) studies.

This section uses a Venn diagram (VD) to assess the relationship between the three approaches for RoB: ranking (RBS), radial bias map (RBM), and radial bias area (RBA) model. The three types of bias employed in the VD process are shown in Figure 19a,c, including low bias (Figure 19a), moderate bias (Figure 19b), and high bias (Figure 19c).The number of studies in low bias (out of 28 studies) for RBM, RBA, and RBS was three (10%) [48,58,62], eight (28%) [45,49,50,57,60,61,64,69], and four (14%) [24,48,65,67] respectively, whereas the number of studies under low bias (out of 60 studies) for RBM, RBA, and RBS was seven (12%) [58,75,79,81,82,86,87], twelve (20%) [28,65,77,84,85,89,90,91,92,93,94,111], and seven (12%) [24,48,55,58,59,62,67], respectively. Out of 28 and 60 studies, respectively, 2 [48,62] and 1 [58] studies fell within the intersection of RBS and RBM, whereas no shared studies were discovered under the intersection of (RBA, RBM, RBS), (RBA, RBM), and (RBA, RBS) in either study.

The number of studies in moderate bias (out of 28 studies) for RBM, RBA, and RBS was three (10%) [53,54,55], nine (32%) [47,51,53,54,56,62,68,71,72], and eleven (39%) [44,46,47,53,54,55,56,57,58,71,99], respectively. The number of studies that fell under the intersection of (RBA, RBM, RBS), (RBA, RBS), and (RBM, RBS) was two, three, and one, respectively, whereas no common studies were found under intersection of (RBA, RBM). On the other hand, the number of studies under moderate bias (out of 60 studies) for RBM, RBA, and RBS was 9 (15%) [43,69,74,78,80,83,95,96,98], 20 (33%) [43,49,57,61,64,69,73,74,78,80,82,83,86,87,88,95,98,120,160,161], and 18 (30%) [44,45,46,47,50,51,52,53,54,55,59,60,61,71,72,75,76,96], respectively. The number of studies that fell under the intersection of (RBA, RBS), (RB, MRBS), and (RBA, RBM) was two, one, and eight, respectively, whereas no common studies were found under the intersection of (RBA, RBM, RBS). 

The number of studies in high bias (out of 28 studies) for RBM, RBA, and RBS was 22 (78%) [24,44,45,46,47,49,50,51,56,57,59,60,61,63,64,66,67,68,69,70,71,72], 11 (39%) [24,44,46,48,52,55,58,59,62,67,99], and 13 (46%) [45,49,50,51,52,57,60,61,64,68,69,70,100], respectively. The studies that fell under the intersection of (RBA, RBM, RBS), (RBA, RBM), and (RBM, RBS) was two, five, and six, respectively, whereas no common studies were found under the intersection of (RBA, RBS). Moreover, the number of studies under high bias (out of 60 studies) for RBM, RBA, and RBS was 44 (73%) [24,28,44,45,46,47,48,49,50,51,52,53,54,55,56,57,59,60,61,62,64,65,66,67,68,69,70,71,72,73,74,77,84,85,88,89,90,91,92,93,94,97,100,111], 28 (46%) [24,43,45,46,47,48,50,51,52,53,54,55,56,58,60,66,67,68,70,71,72,75,79,81,96,99,100,162], and 35 (58%) [28,43,49,56,57,64,65,68,69,70,73,76,77,78,79,80,81,82,83,84,85,86,87,88,89,90,91,92,93,94,95,97,98,111,120], respectively. The number of studies categorized under the intersection of (RBA, RBM RBS), (RBA, RBM), (RBA, RBS), and (RBM, RBS) was 3, 20, 1, and 18, respectively.

The cut-off for the bias can be stratified into three categories: low bias, moderate bias, and high bias. With the change in the cut-off values, the studies can be categorized into generalized studies. Generalized studies are more reliable studies because here, the AI system yields higher accuracy on the unseen datasets. Unseen datasets are the datasets that are not part of the training protocol. In unseen analysis, the system is trained using data from hospital A, and prediction is carried out on the test data from hospital B. A higher accuracy of the unseen analysis proves that the system is more reliable. For the low-bias systems, the generalizability is better than that of the high-bias systems.

In summary, Table 2 below shows the characteristics of how the generalizability and the reliability strengths are depicted with the type of bias.

Generally, bias can be integrated into AI-model at various stages of development and deployment. The various phases of development at which bias can be into model is pre-processing stage, in-processing stage and post-processing stage [163,164,165,166]. Bias in data collection and gathering are primarily reason for pre-processing bias, algorithmic design causes the bias during in-processing stage and the training and model deployment leads to bias during post-processing stage. In order to mitigate bias at various stages of AI-model, critical AI-parameters are chosen which not only enhance the model discriminability but also improve the learning process during model training [167,168,169,170,171,172]. Model outcomes should be explainable and interpretable to ensure its interoperability and generalizability. The model design is black box to the end user. However, the model should be explainable to identify the reasons for AI-bias in the system. The explainable model is helpful in identifying the possible solutions to mitigate bias and generate sophisticated model architecture [169,173,174].

## 6. Explainability in AI

Because of the fact that DL programmers routinely outperform humans at tasks like recommendation systems, voice, and image recognition, among many others, they have gained considerable attention. However, these applications are neither dependable nor understandable. The fact that DL models are intangible and challenging-to-comprehend black boxes with complicated underlying mechanisms is a serious issue. They lack justification for their decisions and predictions, which makes it impossible for people to believe them. These problems are resolved with explainable AI (XAI) [175,176,177,178,179,180]. Models for machine learning operate in a “black box”, which means they can only forecast the outcomes and cannot answer “why family” questions like “Why do you act that way?”, “Why should I believe you?”, “Why is there nothing else?”, “When do you achieve success?” “Why are you failing?”, and many others. 

Explainability in AI can help with (i) the AI model’s debugging, (ii) the outcome’s validation, and (iii) providing a visual justification for the AI model’s classification of the image. Although several studies have recently been published on XAI, the IVUS scan of coronary arteries applications for wall segmentation using UNet has been the subject of only a few investigations. 

When establishing a relationship, correlation, or links between the CVD risk stratification of various clinical outcomes, XAI is even more crucial. This is due to two factors: (a) the fact that XAI was first introduced to the computer vision market in 2015—less than seven years ago—and (b) the lack of integration between some tools, such Shapley additive explanations (SHAP) and UMAP, and DL packages. For location-based data, LIME [181] and SHAPLEY [182] have served the purpose, whereas heatmaps were employed for the image data. 

When we need to validate the outcome, the necessity for XAI is increasingly critical. For instance, in an IVUS scan, we might need to spot any lesions or other IVUS scan artifacts in the plaque-cut regions that are sick. Here is the XAI requirement that yields the desired outcome. We thus employ a heatmap visualization method for an explainable AI model to achieve this. 

There are many characteristics of the lesions namely texture, contrast, intensity variation, and density alteration [183]. Gradient-weighted class activation map (or “Grad-CAM”) was recently designed to reconstruct the color map of the lesion area. Grad-CAM utilizes as the name says the gradient of the target to predict a coarse localization that displays the color heatmap in the form of images of lesions and control region. Whether they entail vascular or non-vascular applications, medical imaging applications must all be understandable and easy to interpret. Thus, UNet with explainability (XAI) offers fresh opportunities in the fields of vascular and non-vascular medicine. 

**Figure 20:**
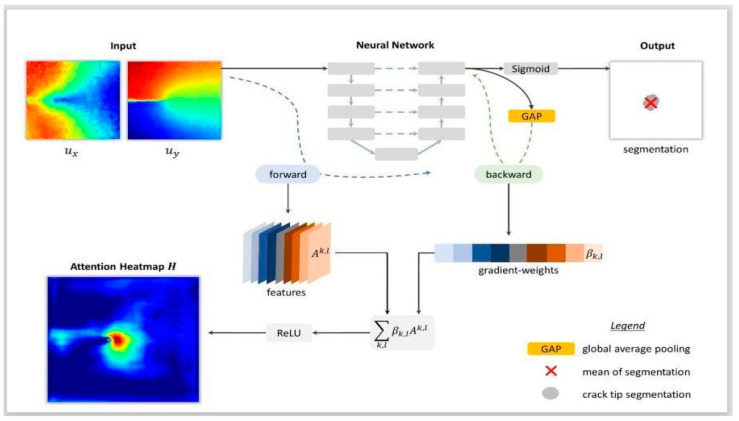
Deep neural network visualization using the GradCAM approach [184].

As shown in Figure 20, Malching et al. [184] proposed a model parallel net. At the point of contention in the parallel net design, the original UNet and fully connected neural network (FCNN) were joined. UNet was used for crack tip segmentation, while an FCNN regressor was used for crack tip position. To examine interpretability, the authors used the Grad-CAM interpretability approach. The forward input data pass of the neural network was used to acquire internal features, which were then weighted to aggregate the backward pass’s average pooled gradients. 

## 7. Pruning in Wall Segmentation of IVUS Scan 

In general, by chopping or deleting any of its weights that are redundant, have little training value, or do not contribute to the design of the loss function, a deep neural network (DNN) can minimize its size using the optimization technique of pruning. Pruning the network [162,185,186,187] can assist in preventing the DNN from overfitting during training. Further, if pruning of channels that share the same features of the image is conducted, then the storage required drastically decreases. 

The pruning methods can be either weight pruning, channel pruning, or hybrid pruning. The weight pruning approach removes the redundant weight and only keeps weights that contribute to the result [188,189,190,191,192], whereas in channel pruning, unnecessary channels are removed from the feature images [193,194,195,196,197,198,199,200]. A pre-trained teacher network architecture is given, and the objective of hybrid pruning [99,201,202] is to seek and identify the shortest network model from that architecture while preserving the best level of accuracy. In comparison to the teacher network, the student network is streamlined and decreased [203].

Neural network pruning can be applied to the IVUS scan of coronary artery wall segmentation using the UNet-based deep learning approach in order to optimize the segmentation architecture design. We can improve the UNet-based IVUS segmentation by cutting the parameter size (total number of training parameters during the supervised training system design). Sometimes, some techniques prune the layers of UNet (by truncating the standardized five layers of Ranneberger), and such pruning is called automated mini UNet (AM-UNet) [204], where the UNet size is decreased by cutting some layers of the UNet, and in this way, the computational complexity of the architecture decreases and also provides good accuracy. There is another pruning method called half-UNet, where the UNet is chopped into half by removing all the decoders and making sure only one decoder remains so that all the skip connections add up using ADDER to make the output; such a system is called half-UNet [205]. 

**Figure 21:**
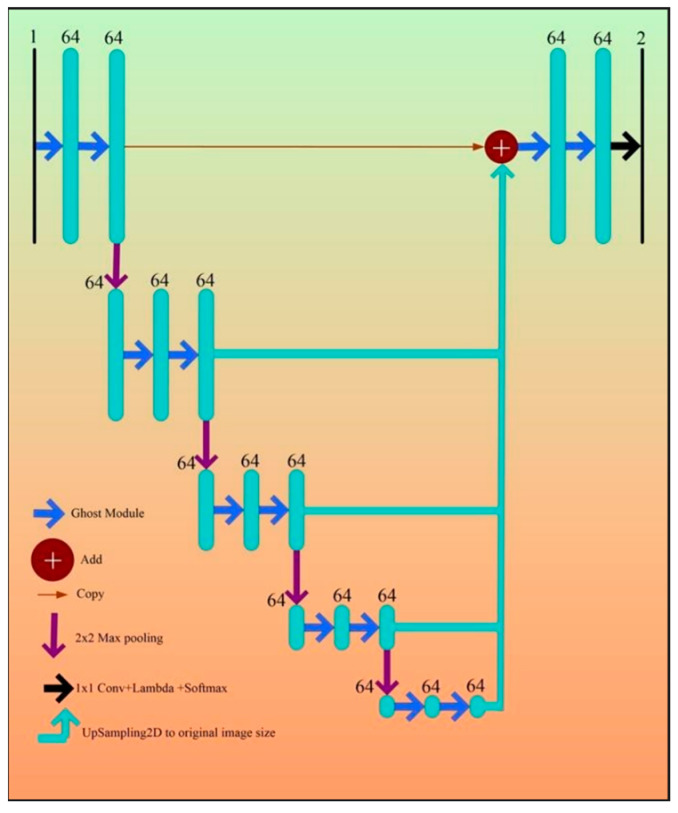
The half-UNet building’s structure. The number of feature map channels is given above the rectangles.

Because it primarily streamlines the feature fusion portion, we here advise using a half-UNet (Figure 21) for pruning-based coronary wall segmentation in IVUS images. Half-UNet decreases network complexity by combining channel numbers, utilizing full scale feature fusion, and implementing ghost module [205]. 

Evolutionary techniques are now employed to enhance training by reducing the number of parameters, including (i) the whale optimization (WO) algorithm, genetic algorithms (GA) [206,207], particle swarm optimization (PSO) algorithm, and differential evolution algorithm (DEA). 

The deep learning networks can be optimized using the four evolutionary algorithm (EA) methods. These are as follows:

Differential evaluation algorithm (DEA): DE is a reproduction method that makes use of unit vectors to convey distance and orientation data and enhances solutions through evolutionary processes [208,209]. To create new vectors, the procedure involves mutation recombination [210,211]. These algorithms swiftly explore huge design areas while making few assumptions about the underlying optimization problem.

Genetic algorithm (GA): A population of individuals who differ from one another is maintained via the second EA approach, which is known as GA and was inspired by Darwin’s theory of evolution [212]. Survival of the fittest refers to the idea that those who are more adapted to their surroundings have a better chance of living, reproducing, and passing on their traits to subsequent generations. GA produces optimized solutions through the processes of selection, crossover, and mutation [213,214].

Particle swarm optimization (PSO): The third EA approach, known as PSO, was first presented by Kennedy and Eberhart [215] in 1995. It is based on the idea that a flock of birds or fish learns from one another to determine the optimal location to eat [216,217]. In this instance, a random 0 and 1 are used to create the location vector. It is assumed that the place of food corresponds to the vector with the maximum fitness. It has a collection of equations for determining fresh location vectors in subsequent iterations.

Whale optimization (WO): Lastly, inspired by the meta-heuristic optimization algorithm [218] such as WO, was used as an Evolutionary Approach. Due to humpback whale behavior of chasing and encircling its prey in a spiral loop, this algorithm was named as WO [219,220]. The position of the prey was located when the best vector with the highest fitness, as a result, the algorithm converges to that location. The algorithm provided the flexibility to create a new set of equations as new position vectors which represented next iteration.

## 8. Critical Discussion

### 8.1. Principal Findings

The proposed investigation concentrated mostly on UNet methods for wall segmentation in IVUS scans. The distinctive features of the UNet-based systems were addressed together with their architectures. When segmenting the walls of the IVUS imaging using the UNet model for statistical analysis, 30 studies were taken into account. It was determined that the main purpose of the UNet is to automatically segment the walls of IVUS images for coronary artery disease based on input risk variables.

Furthermore, UNet-based systems handled the variation among the risk factors and what was actually happening better than conventional-based systems overall. The UNet model was more widely used, but as the AI methodology improved, a different form of UNet was introduced that exhibited superior segmentation results than the UNet because of the combination of several models. The different UNet variations techniques utilized in the DL framework were UNet VGG16, dual-path UNet, MFAUNet, BCDUNet, UNet deep CNN, 3D-dense UNet, eight-layer UNet, UE-Net, retina UNet, attention UNet, IVUS-Net, and T-Net. The risk-of-bias (RoB) assessment was presented in the DL framework for wall segmentation of IVUS pictures. Out of the 60 publications taken into account for UNet and the traditional technique, the bias classifications included low bias (7 papers), moderate bias (18 papers), and high bias (35 papers). The factors that contributed to the bias were also determined.

The major research contributions of our study are as follows:

In-depth analysis of UNet-based deep learning models for wall segmentation in IVUS scans: This covers the state-of-the-art methods used for the characterization of the walls of the IVUS scans. Comparison of conventional vs. UNet-based deep learning methods for wall segmentation in IVUS scans: This comparative table consists of 26 columns corresponding to the attributes used in the comparison. Sixty studies were used when analyzing these 60 studies. The PRISMA method was used for selection of the references and classifying them into correct bins. Statistical analysis was adopted after analyzing all the studies. Three kinds of bias techniques for AI methods were used for analyzing the IVUS-based wall segmentation. Explainability of AI methods was used for IVUS-based segmentation. Pruning techniques for large AI models were used for IVUS segmentation. We benchmarked our study against the previous studies used for IVUS segmentation. We discussed the unsupervised solutions for IVUS segmentation. Possible new UNet-based methods for IVUS wall segmentation were detailed.

### 8.2. Benchmarking

The benchmarking table for IVUS picture segmentation using AI (ML or DL), which consists of fourteen review papers [160,161,221,222,223,224,225,226,227,228,229,230,231], is shown in Table 3. Each relevant study from row R1 to row R14 has eleven attributes (columns C1 to C11) listed in the table. The studies (C1), year of the study (C2), AI specification of the study (C3), objective of the study (C4), use of the PRISMA model (C5), statistical classification role (C6), field of application (C7), architectural classifications of the study (C8), performance or not of bias analysis in the study (C9), number of studies used (C10), and finally, the total number of citations used in the study (C11) were the eleven attributes that were addressed. Row R1, rows R3 to row R5, row R7, and rows R9 to row R11 show the studies that applied both ML and DL methods, whereas rows R2, R6, R12, and R14 show the research that used DL techniques. Segmenting the plaque, risk assessment or classification, detection, and comparison of ML or DL approaches were the goals. Six studies used the PRISMA model as a framework. Six research performed the statistical categorization; other studies did not demonstrate the classification based on statistics. Heart failure, stroke, CKD, RA, myocardial infarction (MI), myocardial perfusion (MP), and CAD were the applications.

### 8.3. A Special Note on Comparison of the Latest Deep Learning Solution vs. UNet-Based Models

For the partition of lumen, vessel, and plaque volume in IVUS, Bass et al. [232] used the ML model. The authors compared the ML-based strategy with the laboratory core (LC) reading-based method for measuring percent atheroma volume (PAV). By taking the 10 mm segments, the gold standard of PAV was at baseline 52.31% and was at follow-up 49.42%. Using the ML solution, the PAV at baseline was 51.55%, while during the follow-up, it was 47.81%. The authors showed the change was <4% with a *p*-value < 0.001. 

In a different study by Arora et al. [233], the authors offered a reference cohort with a larger sample size that was obtained utilizing different transducer frequencies while taking into account complicated and variable lesions. Further, the study showed the gold-standard tracings that are vital for benchmarking the automated methods and during the supervised learning. 

The authors of a more recent study by Blanco et al. [234] created a two-stage paradigm for vessel contours (MA borders) and lumen (LI borders), where stage I involved preliminary segmentation using deep neural network (DNN), and stage II involved a Gaussian process-based (GP) model, similar to the method in [235,236]. The cohort consisted of 160 patients, out of which 100 patients (8427 frames) were used for training, 30 (2583 frames) were used for validation, and 30 (2425 frames) were used for testing (prediction). The authors computed standardized metrics for performance evaluation, namely Dice similarity and Jaccard index, yielding a value of 0.913 [CI: 0.882,0.935] and 0.940 [CI: 0.917,0.957], respectively. 

In study [237], bilateral collaboration learning (BCL) is suggested for vessel contour detection (VCD) in intracoronary pictures. By explicitly splitting the label space and modelling transferable features from both the inter- and intra-domain, it obtained domain-invariant features for bilateral knowledge transfer. Additionally, the BCL instantiated it. For each modality, the BCL extracted domain-invariant features as auxiliary data to enhance contour detection performance. Additionally, it improved the encoder’s capacity to encode high-level semantics data by using transformer architecture. Numerous trials showed that the BCL is more efficient than and superior to the most recent single-modality and cross-modality VCD techniques.

In the study [238], by combining active learning and assisted annotation, the authors presented a unique framework for evaluating segmentation quality that can significantly lessen the annotation effort required for both image selection and annotation. With the help of the probability attention module (PAM), their two-branch network can concurrently learn the parameters for segmenting images and spot segmentation mistakes. Their method successfully reduced the required size of the training set by choosing only the image data that the segmentation quality assessment (SQA) module identified as potentially having larger regions of incorrect segmentation.

A general post-processing segmentation system for IVUS images was developed that computed lumen and EEM borders [239]. For extracting the lumen boundaries in temporal framework an exclusive set of context-based feature encoder was used. Further, for high resolution segmentation, the authors used a selective transformer recurrent UNet. For difficult areas of segmentation, the authors used inference-based segmentation. Finally, for the effective framework, temporal constraint and fusion model was adopted.

### 8.4. A Short Note on UNet and Its Ability 

UNet architecture, which was first released in 2015, has completely revolutionized the field of deep learning. The convolutional neural network UNet’s function is to segment biological pictures. The network architecture was extended and modified from a fully convolutional network’s initial design to handle fewer training images and deliver more accurate segmentation. Utilizing this UNet architecture, segmenting images in sizes of 512 × 512 can be accomplished rapidly on a modern GPU.

There have been many variants and modifications of this architecture due to its phenomenal success. These variants are UNet VGG16, dual-path UNet, MFAUNet, BCDUNet, UNet deep CNN, 3D-dense UNet, eight-layer UNet, UE-Net, retina UNet, attention UNet, IVUS-Net, and T-Net. The purpose of UNet is to gather both the localization and context features. This process is successfully completed by the type of architecture that is built. The main objective of the procedure is to produce outputs with higher resolution on the inputs made by using successive contracting layers that are immediately followed by upsampling operators.

### 8.5. A Special Note on Machine Learning

The ML algorithms can learn and accurately become smarter over time due to the larger (augmented) and variable data sizes. This is because the supervised training model uses the observation data along with the labelled (gold standard or ground truth) datasets. The gold standard act is supervision to the training model, which can be linear or non-linear in nature. When the ML learning model over-learns, then it is under the memorization phase, and the model should be more generalized. Thus, there is always a challenge for the ML system to generalize vs. memorize. While the ML system can learn, the ability of the ML system to perform well also depends upon the feature extracted and the feature selected for training the ML system. Thus, the power of ML can be used for the characterization of the problem [240].

### 8.6. A Special Note on the Differences between Machine Learning (ML) and Deep Learning (DL) Features 

ML features: ML features are the features that are computed manually. They are typically statistical in nature but for the specific regions of the image. The region of interest (ROI) is also computed either manually or semi-automatically in the image. Once the ROI is computed, the statistical feature is extracted for these regions. These regions have different characteristics that can distinguish different types of classes, namely control, mild, moderate, or high risk (or high severity regions). These ML-derived features are typically statistical in nature, such as contrast, moments, frequency domain analysis, signal-based analysis [241,242,243], and local binary patterns (LBP) [244]; texture features based on first-order statistics, gray level co-occurrence matrix, and run length matrix [245]; gray-scale features based on stationary wavelet transform [246]; and higher-order spectra (HoS) [247]. 

Note that these features have different strengths for different kinds of disease classification paradigms. The magnitude of these features is thus the driving force for the ML model classifiers to learn about the characteristics of the classification process. The challenge in ML-based classification is the process by which the features are computed, trained, and applied to transform the test feature for the prediction of the test label. Due to the ad hoc nature of the feature extraction process, the manual application of these features for the generation of the training-based model puts an extra burden on the classifiers. Further, note that these features are sorted out for the best training model generation. In summary, the (i) feature generation, (ii) feature reduction, (iii) feature application for generation of the training-based model, and (iv) application of the trained model to the test features to predict the test labels are almost entirely done manually.

Deep learning features: On the contrary, the power-automated iterative process of convolution followed by max pooling allows for automated feature extraction [117,139]. Following the feature extraction, the weight transformation takes place in forward and backward propagation using neural networks (NN), allowing a powerful and effective paradigm for classification [248]. Since NN provides the flexibility of increasing the number of layers, the refinement of the features is all automated.

Note that deep learning systems are complex to design due to the number of layers in the AI system [145]. The feature extraction process causes a more complex design of DL systems compared to conventional systems. The number of operations that AI systems must undergo is large. This is due to the large number of neurons in the neural network (NN) design [40]. Further, the forward and backward runs in the NN make the number-crunching process complex. This can even become more challenging if the batch size is large. Typical batch sizes can range from 8 to 16 to 24, based on the hardware’s ability, such as the RAM and clock speed of the system. Although this is challenging in DL systems, conventional systems can also face challenges if the number of iterations is large. This can be said of the “level set functions”, which require a greater number of iterations to for the boundary curves to settle at the correct edge of the interfaces. One of the challenges in the DL system is the hardware constraint. The complexity of DL architectures derives from the large number of layers that make up the DL architecture; it is therefore important to use a GPU cluster, which has the capacity to process a large number of instructions per second. This increases the cost of the AI system design. One of the biggest challenges in the DL system is the need for the optimization of DL algorithms, and this requires the fine tuning of the hyper-parameters. The items included in this are as following: learning rate, batch size, epoch per iteration, iteration count, optimizer scheme (such as adaptive moment estimation (ADAM), stochastic gradient descent (SGD), and interaction with the quality control system), and learning rate. The decision of the necessary amount of samples is one of the main issues and challenges in deep learning models. The data sample not only needs to be large in size, but it needs to consist of quality datasets. Thus, one needs a strong quality control system through actions such as augmentation, balancing, filling values, scaling of the values, normalizations, etc.

### 8.7. Pros and Cons of Conventional and AI Systems

While conventional systems have poor performance and are ad hoc in nature [130,224], AI systems also offer several challenges, such as complex design due to large number of NN layers [249,250], increased number crunching due to forward and backward propagation, and restrictions of limited batch sizes [251]. The other challenges include the hardware and its cost requirements due to the GPU cluster need [252], the need for optimization and hyper-parameter tuning [41], and finally, the need for data size requirements [151,253,254,255]. 

### 8.8. Advantage of the UNet Architecture

UNet has been extensively utilized in the computer vision industry for image-based segmentation tasks. UNet consists of multiple parts, including the bottleneck layer, skip-connection, encoder, and decoder. These parts can be combined in a number of ways to create a strong UNet system that can be used for both vascular and non-vascular tasks [39].

### 8.9. A Short Note on Unsupervised Paradigms 

Unsupervised learning refers to a machine learning method that uses training datasets without constantly checking the models. Instead, the model analyzes the incoming data to extract patterns and insights that were previously concealed. The branch of machine learning where training of the model does not utilize the gold standard can be categorized into unsupervised learning. Sometimes, in unsupervised learning the training model uses ad hoc labels as gold standard which can therefore be used as training. These ad hoc labels can be derived from the unsupervised architecture itself and therefore, can be reused as gold standard. Thus, unsupervised learning is a kind of supervised learning in nature. In the unsupervised learning protocol, three sets of operation typically happen, namely, revealing of the structure, arranging and presenting in the concise form of the dataset. Even though unsupervised learning sounds challenging, it tries to mimic the human learning based on their experiences, thereby, cloning typical AI. Since, the unsupervised learning involves both unlabeled and uncategorical paradigms, therefore becomes more important during their design. There are several popular unsupervised learning techniques includes principal component analysis, neural networks, k-means clustering, KNN, hierarchical clustering, and independent component analysis.

### 8.10. Strengths, Weakness, and Extensions 

The major advantage of this study was the selection and collection of the repository of studies which led us to better understanding of IVUS-based wall segmentation using UNet-based paradigm. The current research offered insight into the structural variances of the UNet-based paradigm on 29 different types of AI features. This study offers a ranking-based method for categorizing ML experiments into three groups (LB, MB, and HB). Also, we analytically showed that studies using UNet had less bias than studies that did not use UNet. Overall, our research established the connection between the LB, MB, and HB distributions and described the AI-attributed behavior throughout 30 UNet and 30 non-UNet investigations. 

Even though the outcomes of previous studies were positive and promising, it lacks clarity and evidence of the missing data. Numerous advanced-AI groups have not participated due to lack of resources and funding and ability to share proprietary datasets. Not every study included all of the attributes listed in the benchmarking table. By evaluating in-depth a greater number of attributes, the study can be improved for more accurate bias estimations. Further, fusion techniques can be incorporated to improve the wall segmentation.

Recent research suggests CAD risk stratification using surrogate biomarkers such as carotid artery disease [256,257,258,259,260]. In the future, there is a need to understand the vascular implications of COVID-19 on CAD [20,261,262]. Other applications include understanding erectile dysfunction using CAD [221,263]. Implementing attention mechanisms (such as self-attention) can help the model focus on relevant parts of the IVUS images, improving accuracy. Generative adversarial networks (GANs) can be used to generate synthetic IVUS images, which can augment the training dataset and enhance the segmentation model’s performance. Fusion of IVUS data with other imaging modalities like optical coherence tomography (OCT) or angiography can provide complementary information, leading to more accurate segmentation results. Future developments will focus on creating efficient algorithms that can segment coronary artery walls in real-time, enabling immediate feedback to clinicians during procedures.

## 9. Conclusions

This study focused on an in-depth analysis of UNet-based deep learning models for wall segmentation in IVUS scans. The comparative table consists of 26 columns corresponding to the attributes used in the comparison of conventional vs. UNet-based deep learning methods for wall segmentation in IVUS scans and is based on 60 studies. The PRISMA model was adopted for the selection of references and their classification into correct sections. After analyzing all the research, statistical analysis was used. UNet-based architecture is the most powerful paradigm for coronary wall segmentation because of its capacity for automatically obtaining contextual and semantic characteristics despite numerous variations in IVUS scans. The versatility of a UNet-based architecture allows for the provision of UNet variations with different designs for encoders, decoders, skip connections, bottleneck layers, and loss functions. Due to the lack of clinical interface in the studies, there exists a bias in DL models. We demonstrated three kinds of RoB methods, namely ranking, regional, and radial methods. We further showed that these bias methods are potent frameworks for bias estimation in UNet models for CAD wall segmentation in IVUS scans. The low-moderate (LM) and moderate-high (MH) cut-offs, which were found to be 2.9 and 2.5, respectively, divided the 28 DL-based research into three categories: low bias (4 studies), moderate bias (11 studies), and high bias (13 studies). We provided a set of five points as recommendations for reducing the RoB, which include the following: (i) larger cohort size, (ii) superior gold-standard collection, (iii) usage of the tools in clinical settings, (iv) adaptation of different variations of UNet to ensure the superior segmentation outcome, and (v) consistent data collection using multicenter cohorts. The deep learning networks can be optimized using the four evolutionary methods: whale optimization, genetic algorithm, particle swarm algorithm, and differential evaluation algorithm. 

## Figures and Tables

**Table 1:** Characteristics of UNet and conventional systems for CAD.

		A1	A2	A3	A4	A5	A6	A7	A8	A9	A10	A11	A12	A13
SN	Studies	TP	SM	HT	AU	L	EC	DC	SC	LF	PL	DSC	PV	JI
1	He et al. [44]	2	-	-	U	5	3	3	√	√	√	√	-	√
2	Jin et al. [95]	5	-	-	C	-	-	-	-	-	-	-	√	-
3	Ibtehaz et al. [45]	1	-	-	U	5	5	5	√-	√	√	-	-	√
4	Balakrishna et al. [46]	1	-	-	U	5	5	5	√	-	√	√	-	√
5	Kim et al. [48]	1	-	-	U	5	5	5	√	√	√	√	-	√
6	Li et al. [47]	1	-	-	U	5	4	4	√	√	√	√	-	-
7	Chen et al. [49]	1	-	-	U	5	3	3	√	√	√	√	-	-
8	Tong et al. [50]	1	-	-	U	5	3	3	√	√	√	√	-	-
9	Morris et al. [51]	1	-	-	U	5	4	4	√	√	√	√	-	-
10	Zhou et al. [52]	1	-	-	U	5	5	5	√	√	√	√	-	-
11	Milletari et al. [53]	1	-	-	U	5	5	5	√	√	-	√	-	-
12	Szarski et al. [54]	1	-	-	U	5	5	5	√	√	√	-	-	√
13	Vercio et al. [65]	0	-	-	C	-	-	-	-	-	-	-	-	√
14	Yang et al. [55]	1	-	-	U	5	4	4	√	-√	√	-	-	√
15	Shen et al. [56]	5	-	-	U	5	4	4	√	√	-	√	-	√
16	Javorszky et al. [57]	5	-	-	U	5	4	4	√	√	√	-	√	-
17	Momin et al. [58]	5	-	-	U	5	3	3	-	-	√	√	√	-
18	Guo et al. [99]	5	√	√	U	5	4	4	√	√	√	√	√	-
19	Huang et al. [67]	5	-	-	U	5	5	5	√	√	√	√	-	√
20	Jun et al. [100]	0	-	-	U	5	5	5	-	√	√	√	-	-
21	Shi et al. [64]	0	-	-	U	5	5	5	√	√	√	-	-	-
22	Thuy et al. [68]	0	-	-	U	5	4	4	√	√	√	√	-	-
23	Hwang et al. [69]	0	-	-	U	5	5	5	-	-	-	-	-	-
24	Cheung et al. [60]	5	-	-	U	5	5	5	√	-	√	√	-	-
25	Dong et al. [61]	3	-	-	U	5	8	8	√	√	-	-	-	√
26	Pan et al. [70]	0	-	-	U	5	4	4	√	√	√	√	-	√
27	Song et al. [24]	5	-	-	U	5	4	4	√	√	√	√	-	-
28	Shinohara et al. [62]	3	-	-	U	5	5	5	√	-	√	√	√	√
29	Yang et al. [55]	0	-	-	U	5	5	5	√	√	√	-	-	√
30	Xia et al. [71]	0	-	-	U	5	5	5	√	√	√	-	-	√
31	Azad et al. [72]	4	-	-	U	5	4	4	√	-	√	-	-	-
32	Ronneberger et al. [73]	0	-	-	U	5	4	4	√	√	√	-	-	-
33	Bajaj et al. [74]	1	-	√	C	-	-	-	-	√	-	-	√	-
34	Cho et al. [75]	5	-	-	C	-	-	-	-	√	-	-	√	-
35	Araki et al. [26]	2	-	-	C	-	-	-	-	-	-	√	√	√
36	Bajaj et al. [76]	2	-	-	C	-	-	-	-	√	-	-	-	-
37	Fedewa et al. [77]	0	-	-	C	-	-	-	-	-	-	-	-	-
38	Masuda et al. [78]	5	-	-	C	-	-	-	-	√	-	-	-	-
39	Min et al. [79]	1	-	√	C	-	-	-	-	√	-	-	√	-
40	Nishi et al. [80]	2	-	-	C	-	-	-	-	√	√	√	√	-
41	Shin et al. [111]	2	-	-	C	-	-	-	-	-	-	-	√	-
42	Olender et al. [81]	1	-	-	C	-	-	-	-	√	-	-	√	-
43	Zhao et al. [82]	2	-	-	C	-	-	-	-	-	-	-	√	-
44	Bargsten et al. [83]	1	-	-	C	-	-	-	-	√	-	√	-	-
45	Samuel et al. [96]	3	-	√	C	-	-	-	-	√	√	-	-	-
46	Faraji et al. [28]	1	-	-	C	-	-	-	-	-	-	-	-	√
47	Tayel et al. [84]	1	-	-	C	-	-	-	-	-	-	-	-	-
48	Cui et al. [85]	1	-	-	C	-	-	-	-	-	-	-	-	√
49	Harms et al. [86]	5	-	-	C	-	-	-	-	-	-	√	√	-
50	Mishra et al. [87]	0	-	-	C	-	-	-	-	√	√	√	-	-
51	Lin et al. [97]	5	-	-	C	-	-	-	-	-	-	√	√	-
52	Du et al. [98]	0	-	-	C	-	-	-	-	√	-	√	-	√
53	Hwang et al. [69]	2	-	-	C	-	-	-	-	-	-	-	-	-
54	Jodas et al. [88]	0	-	-	C	-	-	-	-	-	-	√	-	√
55	Eslamizadeh et al. [89]	0	-	-	C	-	-	-	-	-	-	-	-	-
56	Sofian et al. [90]	1	-	-	C	-	-	-	-	-	-	-	-	√
57	Cao et al. [91]	3	-	-	C	-	-	-	-	-	-	√	√	√
58	Taki et al. [92]	1	-	-	C	-	-	-	-	-	-	-	-	-
59	Unal et al. [93]	0	-	-	C	-	-	-	-	-	-	-	-	-
60	Zhu et al. [94]	0	-	-	C	-	-	-	-	-	-	-	-	-
		**A14**	**A15**	**A16**	**A17**	**A18**	**A19**	**A20**	**A21**	**A22**	**A23**	**A24**	**A25**	**A26**
**SN**	**Studies**	**HD**	**Val**	**LR**	**BS**	**EPO**	**OPT**	**DA**	**Acc.**	**Pres.**	**RS**	**SN**	**SP**	**CE**
1	He et al. [44]	√	-	-	-	-	-	-	-	-	-	√	√	S
2	Jin et al. [95]	-	-	-	-	-	-	-	√	√	√	√	√	M
3	Ibtehaz et al. [45]	-	-	-	-	√	-	√	√	-	-	-	-	M
4	Balakrishna et al. [46]	-	-	√	√	√	-	√	√	-	-	-	-	S
5	Kim et al. [48]	√	√	√	√	√	-	-	-	-	-	-	-	S
6	Li et al. [47]	-	-	√	√	√	√	-	-	√	-	√	√	S
7	Chen et al. [49]	-	-	-	-	-	-	√	-	-	-	-	-	S
8	Tong et al. [50]	-	-	-	√	√	-	√	-	-	-	-	-	S
9	Morris et al. [51]	-	-	√	√	√	-	√	-	-	-	-	-	S
10	Zhou et al. [52]	-	-	-	-	-	-	-	-	-	-	√	√	S
11	Milletari et al. [53]	√	-	√	√	√	-	√	-	-	-	-	-	S
12	Szarski et al. [54]	√	-	√	√	√	√	√	-	-	-	-	-	S
13	Vercio et al. [65]	√	-	-	-	-	-	-	-	-	-	-	-	S
14	Yang et al. [55]	√	-	√	√	√	√	√	-	-	-	-	-	S
15	Shen et al. [56]	-	-	√	√	√	√	-	-	-	-	-	-	S
16	Javorszky et al. [57]	-	-	-	-	-	√	-	-	-	-	-	-	S
17	Momin et al. [58]	√	-	√	√	√	√	√	-	-	-	-	-	M
18	Guo et al. [99]	√	√	-	-	-	-	√	-	-	-	-	-	S
19	Huang et al. [67]	-	-	√	√	√	√	√	-	-	-	-	√	S
20	Jun et al. [100]	-	-	√	√	√	√	√	-	-	-	-	-	S
21	Shi et al. [64]	-	-	√	-	√	-	-	-	-	-	-	-	S
22	Thuy et al. [68]	-	-	√	-	-	-	√	-	-	-	-	-	S
23	Hwang et al. [69]	-	-	√	√	√	-	-	-	-	-	-	-	S
24	Cheung et al. [60]	-	-	√	-	√	√	-	-	-	-	-	-	S
25	Dong et al. [61]	-	-	√	√	-	√	√	-	-	-	-	-	S
26	Pan et al. [70]	-	-	-	-	-	-	-	-	-	-	-	-	S
27	Song et al. [24]	-	-	√	√	√	√	-	-	√	√	-	-	S
28	Shinohara et al. [62]	-	-	√	√	-	√	√	√	√	√	-	-	S
29	Yang et al. [55]	√	√	√	√	√	√	√	-	-	-	-	-	S
30	Xia et al. [71]	-	-	√	√	√	√	√	-	-	-	-	-	S
31	Azad et al. [72]	-	-	-	-	√	-	-	√	-	-	√	√	S
32	Ronneberger et al. [73]	-	-	-	√	-	-	√	-	-	-	-	-	S
33	Bajaj et al. [74]	-	-	√	√	√	-	-	√	√	-	-	-	S
34	Cho et al. [75]	-	√	√	-	√	√	√	√	-	-	√	√	S
35	Araki et al. [26]	-	-	-	-	-	-	-	-	-	√	-	-	S
36	Bajaj et al. [76]	-	-	√	√	√	-	√	-	-	-	-	-	S
37	Fedewa et al. [77]	-	-	-	-	-	-	-	-	-	-	-	-	S
38	Masuda et al. [78]	-	-	√	-	-	-	√	√	-	-	-	-	S
39	Min et al. [79]	-	√	√	-	-	-	√	√	√	-	√	√	S
40	Nishi et al. [80]	-	-	√	√	√	√	-	-	-	-	-	-	S
41	Shin et al. [111]	-	-	-	-	-	-	-	-	-	-	-	-	S
42	Olender et al. [81]	-	-	√	√	√	-	-	√	√	-	-	-	S
43	Zhao et al. [82]	-	√	-	-	-	-	√	√	√	√	-	-	S
44	Bargsten et al. [83]	-	√	√	√	√	√	-	-	-	-	-	-	S
45	Samuel et al. [96]	-	-	√	√	√	-	√	√	-	-	√	√	M
46	Faraji et al. [28]	√	-	-	-	-	-	-	-	-	-	-	-	S
47	Tayel et al. [84]	-	-	-	-	-	-	-	√	-	-	-	-	S
48	Cui et al. [85]	-	-	-	-	-	-	-	-	-	-	-	-	S
49	Harms et al. [86]	√	√	√	√	√	√	-	-	-	-	-	-	S
50	Mishra et al. [87]	-	√	√	√	√	-	√	-	-	-	-	-	S
51	Lin et al. [97]	-	-	-	-	-	-	-	-	-	-	√	√	M
52	Du et al. [98]	√	-	-	√	√	√	√	-	-	-	-	-	M
53	Hwang et al. [69]	-	√	-	-	-	-	-	-	-	-	√	√	S
54	Jodas et al. [88]	√	-	-	-	-	-	-	-	-	-	-	-	S
55	Eslamizadeh et al. [89]	-	-	-	-	-	-	-	√	-	-	-	-	S
56	Sofian et al. [90]	√	-	-	-	-	-	-	-	-	-	-	-	S
57	Cao et al. [91]	-	-	-	-	-	-	-	-	-	-	-	-	S
58	Taki et al. [92]	-	-	-	-	-	-	-	-	-	-	-	-	S
59	Unal et al. [93]	-	-	-	-	-	-	-	-	-	-	-	-	S
60	Zhu et al. [94]	√	-	-	-	-	-	-	-	-	-	-	-	S

TP, total patients; SM, smoking; HT, hypertension; AU, architecture used; L, layers; EC, encoder; DC, decoder; SC, skip connection; LF, loss function; Pool, pooling; DSC, Dice similarity coefficient; SN, sensitivity; SP, specificity; JI, Jaccard index; HD, Hausdorff distance; Acc, accuracy; PV, *p*-value; Pres, precision; RS, recall score; Val, validation; LR, learning rate; BS, batch size; Epo, epochs; OPT, optimization; DA, data augmentation; CE, clinical evaluation; U, UNet; C, conventional; S, single center; M, multicenter; √ implies that a particular attribute (column) was implemented in that study (row).

**Table 2:** Types of Bias.

Bias Type	Generalizability	Reliability
High bias	Low	Low
Moderate bias	Moderate	Moderate
Low bias	High	High

**Table 3:** Benchmarking Table.

	C1	C2	C3	C4	C5	C6	C7	C8	C9	C10	C11
SN	Studies	Year	AI Spec.	Obj.	PRISMA	Stat. Classn	Application	Arch. Classn	RoB	# of Studies	T. Citations
1	Jamthikar et al. [222]	2021	ML/DL	Risk assessment	P	P	CVD, CKD	P	O	120	120
2	Lin et al. [160]	2021	DL	Risk assessment	O	O	CAD	P	O	18	58
3	Faizal et al. [223]	2021	ML/DL	Risk prediction	O	O	CVD	P	O	139	139
4	Biswas et al. [224]	2021	ML/DL	Segmentation	O	P	CVD	P	O	O	163
5	Saba et al. [225]	2021	ML/DL	Comparison	P	P	CVD	P	O	229	229
6	Hinai et al. [226]	2021	DL	Detection	P	P	MI	P	O	12	48
7	Yasmin et al. [227]	2021	ML/DL	Detection	O	O	Heart failure	P	O	22	128
8	Jamthikar et al. [228]	2020	ML	Risk assessment	P	P	CVD	P	O	120	120
9	Monti et al. [229]	2020	ML/DL	Detection	O	O	CAD, MP	P	O	O	40
10	Saba et al. [230]	2019	ML/DL	Risk assessment	P	O	CVD, stroke	P	O	111	111
11	Khanna et al. [221]	2019	ML/DL	Risk assessment	O	O	RA, CVD	P	O	150	150
12	Krittanawong et al. [231]	2019	DL	Comparison	O	O	CVD	P	O	20	105
13	Banchhor et al. [161]	2018	ML	Stratification	O	O	CVD	P	O	153	153
14	Proposed study	2022	DL	Segmentation	P	P	CAD	P	P	105	105

SN, serial number; AI Spec., AI specialization; Obj., objective; Stat Classn, statistical classification; Arch. Classn, architectural classification; #, number; T. Citations, total citations; CVD, cardiovascular diseases; CKD, chronic kidney diseases; MI, myocardial Infraction; MP, myocardial perfusion; CAD, coronary artery diseases; and RA, rheumatoid arthritis.

## Data Availability

Data will be made available on reasonable request.

## Supplementary Materials

The following supporting information can be downloaded at: https://www.mdpi.com/article/10.3390/jcdd10120485/s1, Table S1: Non-UNet/conventional method of DL systems; Table S2: UNet method of deep learning systems. References [24,28,43,44,45,46,47,48,49,50,51,52,53,54,55,56,57,58,59,60,61,62,64,65,66,67,68,69,70,71,72,73,74,75,76,77,78,79,80,81,82,83,84,85,86,87,88,89,90,91,92,93,94,95,96,97,98,100,111,120] are cited in supplementary materials.

## Appendix A. Ranking Tables

**Table A1:** Ranking of IVUS-based studies that uses UNet architectures, utilizing the scores obtained from the bias model.

Studies	Mean	Cumulative Mean	Rank
Shinohara et al. [62]	3.6	3.6	1
Huang et al. [67]	3.3	6.9	2
Song et al. [24]	3.1	10	3
Li et al. [47]	3.0	13	4
Momin et al. [58]	2.9	15.9	5
Yang et al. [55]	2.9	18.8	6
Guo et al. [99]	2.9	21.7	7
Kim et al. [48]	2.8	24.5	8
Szarski et al. [54]	2.8	27.3	9
Balakrishna et al. [46]	2.7	30	10
Milletari et al. [53]	2.7	32.7	11
Yang et al. [66]	2.7	35.4	12
Shen et al. [56]	2.6	38	13
Xia et al. [71]	2.6	40.6	14
He et al. [44]	2.6	43.2	15
Morris et al. [51]	2.5	45.7	16
Jun et al. [59]	2.4	48.1	17
Dong et al. [61]	2.4	50.5	18
Tong et al. [50]	2.3	52.8	19
Ibtehaz et al. [45]	2.3	55.1	20
Zhou et al. [52]	2.3	57.4	21
Cheung et al. [60]	2.3	59.7	22
Thuy et al. [68]	2.1	61.8	23
Jávorszky et al. [57]	2.1	63.9	24
Chen et al. [49]	2.0	65.9	25
Shi et al. [64]	2.0	67.9	26
Pan et al. [70]	2.0	69.9	27
Hwang et al. [69]	1.6	71.5	28

**Table A2:** Ranking of IVUS-based studies that uses non-UNet architectures, utilizing the scores obtained from the bias model.

Studies	Mean	Cumulative Mean	Rank
Cho et al. [75]	2.10	2.10	1
Samuel et al. [96]	2.10	4.20	2
Olender et al. [81]	2.07	6.27	3
Min et al. [79]	1.97	8.24	4
Harms et al. [86]	1.83	10.07	5
Bajaj et al. [74]	1.72	11.79	6
Bajaj et al. [76]	1.69	13.48	7
Jin et al. [95]	1.69	15.17	8
Nishi et al. [80]	1.69	16.86	9
Mishra et al. [87]	1.62	18.48	10
Masuda et al. [78]	1.59	20.07	11
Du et al. [98]	1.52	21.59	12
Zhao et al. [82]	1.38	22.97	13
Bargsten et al. [83]	1.34	24.31	14
Lin et al. [97]	1.24	25.55	15
Araki et al. [43]	1.17	26.72	16
Cao et al. [91]	0.97	27.69	17
Fedewa et al. [77]	0.97	28.66	18
Hwang et al. [120]	0.93	29.59	19
Vercio et al. [65]	0.79	30.38	20
Taki et al. [92]	0.76	31.14	21
Jodas et al. [88]	0.72	31.86	22
Cui et al. [85]	0.66	32.52	23
Tayel et al. [84]	0.66	33.18	24
Faraji et al. [28]	0.66	33.84	25
Shin et al. [111]	0.66	34.50	26
Sofian et al. [90]	0.62	35.12	27
Eslamizadeh et al. [89]	0.59	35.71	28
Zhu et al. [94]	0.45	36.16	29
Unal et al. [93]	0.41	36.57	30

## Appendix B

**Table A3:** Acronym table.

SN	Acronym	Definition	SN	Acronym	Definition
1	AGs	Attention gates	33	LATM	Location-adaptive threshold method
2	AI	Artificial intelligence	34	LI	Lumen-intima
3	Bi-GRU	Bidirectional gated recurrent unit	35	LSTM	Long short-term memory
4	BN	Batch normalization	36	MA	Media-adventitia
5	CAD	Coronary artery disease	37	MFAUNet	Multi-scale feature aggregated UNet
6	CCTA	Coronary CT angiography	38	MI	Myocardial infarction
7	CKD	Chronic kidney diseases	39	ML	Machine learning
8	CNN	Convolutional neural network	40	MRI	Magnetic resonance imaging
9	CSA	Cross-sectional area	41	NB	Naïve Bayes
10	CT	Computed tomography	42	NIRS	Near-infrared spectroscopy
11	CVD	Cardiovascular disease	43	PCA	Principle component analysis
12	DCNN	Deep convolutional neural network	44	PCI	Percutaneous coronary intervention
13	DL	Deep learning	45	PReLU	Parametric rectified linear unit
14	DPUNet	Dual-path UNet	46	PSO	Particle swarm optimization
15	EAT	Epicardial adipose tissue	47	RA	Rheumatoid arthritis
16	EEM	External elastic membrane	48	RF	Random forest
17	EREL	Extremal area of extremum level	49	RNN	Recurrent neural network
18	FAM	Feature aggregated module	50	RoB	Risk of bias
19	FCM	Fuzzy c-means	51	RPN	Regional proposal network
20	FCNN	Fully convolutional neural network	52	RRS	Random radius symmetry
21	FNs	False negatives	53	RUS	Random undersampling
22	FPs	False positives	54	SATM	Scan-adaptive threshold method
23	GA	Genetic algorithm	55	SDL	Solo deep learning
24	GAN	Generative adversarial network	56	SVM	Support vector machine
25	GT	Ground truth	57	TL	Transfer learning
26	GVF	Gradient vector flow	58	US	Ultrasound
27	HDL	Hybrid deep learning	59	VGG	Visual geometric group
28	HMRF	Hidden Markov random field	60	VH	Virtual histology
29	IMTV	Intima-media thickness variability	61	VSSC-Net	Vessel-specific skip chain network
30	IVOCT	Intravascular optical CT	62	WO	Whale optimization
31	IVUS	Intravascular ultrasound	63	XAI	Explainable AI
32	KNN	K-nearest neighbors	64	XCA	X-ray coronary angiography

Fuzzy approach

The fuzzy method works incredibly well for image segmentation. The most significant benefit of fuzzy c-means clustering is its high identification rate and low false-location rate. However, noise affects the fuzzy c-means method. The FCM algorithm does not segment images with complex textures, but it works well for images with simple backgrounds and textures and retains more original image information. This ignores spatial knowledge and only detects information on a grey scale. The primary challenge with this approach is the absence of a precise border.

Parametric approach

The snake model for IVUS scan arterial wall segmentation has been covered here. Identification and outlines of the target item for segmentation are the main tasks performed by the model. The snake model is employed in the field of medical imaging to segment a specific area of an image that differs from other areas of the image in a particular manner. A number of difficulties with the snake model technique are addressed in advanced contour approaches, including noise sensitivity and incorrect contour identification in objects with high levels of complexity.

Geometric approach

One type of contour model called geometric active contour (GAC) moves the curve’s points perpendicularly to modify the smooth curve created in the Euclidean design. The points travel at a speed determined by the region’s curvature in the image. Contours are defined by the geometric flow of the curve and the identification of objects in the image. Both internal and external geometric measures in the area of interest are included in geometric flow. Instead of using snakes, a geometric solution is used to identify objects in an image. The level-set functions, which identify the distinct sections of the image for segmentation, play a crucial role in these contour models. The issue with this strategy is that, for the most part, it lacks these inefficiencies; but, because of their complexity, they are challenging to apply.

## Appendix C

**GradCAM**

XAI is a novel method of presenting attributes that emphasizes the salient features of an image have the greatest influence on the model, as opposed to exhibiting individual pixels. The effect of XAI shows the regions with different colors, namely, red, yellow, blue, which are reflected by the AI model identification in the image. According to the colour scheme, XAI uses red to represent the most influential regions, yellow to represent the medium influential region, and blue to represent the least significant aspects. The main advantage of GradCAM is its ability to validate whether the predicted classification or forecasted value are correct. Further it helps in troubleshooting the output of the classification. Explainability has three benefits: it may be used to (i) validate the results, (ii) debug the AI model, and (iii) give a visual explanation of the factors that led the AI model to classify the image in a particular way [40,41,42]. During the explainabilty, GradCAM helps in the visualization of AI prediction and its validation, given the color palette. Grad-CAM helps in getting a localization color map which indicates the lesions of a particular color (preferably red). This is because it utilizes gradients of the target label or class settling into the last convolution layer. To highlight the IVUS lesions, using explainable AI, the Grad-CAM (Equations (A1) and (A2)) depicts the color combination on the input image, given the AI model. Note that, the color maps are generated based on the class probability score. Further note that the process of model loss computation is accordance with the standard prediction cycle. Based on the output from the desired model layer, one can calculate the gradient in terms of model loss.

Lastly, pre-processing (Equation (A3)) is applied to the gradient areas that aid in the prediction, superimposing the heatmap over the initial gray-scale scans. The original image is then superimposed on top of this map.

(A1) $$w_{k}^{c}=\frac{1}{Z}\sum_{i}\sum_{j}\left(\frac{{\partial Y}^{c}}{{\partial A}_{ij}^{k}}\right)$$

(A2) $$w_{k}^{c}=\sum_{i}\sum_{j}a_{ij}^{kc}.\;relu\left(\frac{{\partial Y}^{c}}{{\partial A}_{ij}^{k}}\right)\;$$

$where\;Y^{c}=\sum_{k}w_{k}^{c}.\sum_{i}\sum_{j}A_{ij}^{k}$

(A3) $$L_{ij}^{c}=\sum_{k}w_{k}^{c}.A_{ij}^{k}\;$$

Here, the final score of class *c* is represented by $Y^{c}$, while the global average pool of the large convolution layer is denoted by the symbol, $A^{k}$. Note that the $w_{k}^{c}$ represents estimated weights for the last convolutional layer for the class *c,* while $L_{ij}^{c}$ represents a class-specific saliency map for each spatial location (*i, j*).

## Appendix D

Pruning Training Models

It is crucial to talk about the key characteristics of the three kinds of PAI networks before analysing their similarities and differences. Model pruning in weight pruning is accomplished by QLP [264] architecture using a single sequential pass from the first to the last. To achieve more significant granular pruning, it gradually prunes each layer in multiple rounds. We found that weight pruning produces four robust DNN architectures: ResNet-32, -56, -50, and MobileNet-v1. CIFAR-10 and ImageNet are the two genuine datasets that show sparsity levels ranging from 50% to 98%. The drawback of this model is that it can only be used for unstructured pruning and needs a DNN model that has already been trained. Alternatively, CLIP-Q [265] is a DNN compression algorithm that carries out weight pruning and quantization concurrently with fine tuning. Pruning rate and bit budget are the two hyper-parameters that are used.The authors showed that the compression rates of AlexNet (51×) had improved by 35%, VGG-16 (72×) by 48%, GoogleNet (10×) by 57%, and ResNet-50 (15×) by 139%, in that order. The algorithms’ accuracy and compression rate were examined in the study, but no information was provided on FLOPs (floating-point operations per second), compilation time, or energy usage.

The DeepCompNet [266,267] algorithm demonstrated quantization utilising the density-based clustering algorithm (DBSCAN), compressing neural networks with Z-score in weight pruning, and Huffman encoding. For devices with limited resources, the Z-score method provides a straightforward and useful architecture. Weight distribution can be easily implemented with Z-score to produce a sparse weight matrix for weight pruning. The raw score’s location, or the deviation from the mean, is represented by the Z-score. Z-score can be calculated using the following formula: $Z_{i}=\frac{{(W}_{i}-\beta )}{\alpha },$ where $W_{i}$ is the $i^{th}$ weight of the current layer, and β and α are the mean and the standard deviation of weight vector, respectively. However, the algorithm has limited functionality for the LeNet architecture.

Filter or channel pruning [268] involves pruning redundant and irrelevant filters at the same time in the same iteration. Several pruning algorithms, including VGG-16, ResNet, and MobleNet, were created to trim the CNN models’ filters. In MobileNetv1, the pruning algorithm’s implications resulted in a 67.03% reduction in computing costs and a 45.45% reduction in memory requirements. To improve compression performance and lessen accuracy loss, this technique can also be utilized with KD. On the other hand, the hybrid pruning paradigm RFC-HyPGCN [202] makes use of a sparse feature compressing architecture. The primary advantage of sparse feature compactness was enhancing network efficiency by eliminating zero storage in between layers. Since this kind of pruning increases the computational load, it is useful for action recognition networks that demand high power consumption. In addition to increasing the model’s throughput, this will speeds up processing.

Applications-wise, the hybrid pruning algorithms are employed for action recognition applications, while the weight and channel pruning algorithms are mostly used for picture classification, segmentation, and detection. While hybrid pruning is used to both CNN and GCNs, weight pruning and channel pruning are mostly applied to CNN models alone. We need to provide more pruning approaches that are utilised to condense the DNN model in addition to weight, filter, and hybrid pruning.

## Appendix E. Half-UNet Concepts

Lu et al. [205] introduced Half-UNet, unlike conventional UNet, where the ideas was lower the complexity without trading off the feature extraction process. This process led into three types of innovations. The three concepts were named as follows: (i) unification of channels, meaning that each layer should have the same number of channels; (ii) Using the fundamental “addition operation”, post upsampling, Lu et al. [205] utilized scaled feature maps derived from the encoders (contractual path) leading to full-scale feature fusion, (iii) the bottleneck convolution complexity reduction using a ghost model. After examining the complex nature of the UNet and UNet3+ models, the essence of the channel’s unification was demonstrated. Every downsampling step in these models resulted in a doubling of the channel count. For the uneven number of channels, Lu et al. [205] added a fundamental 3 × 3 convolution operation for every max-pool operation in UNet3+. As the result of unification of the channel numbers, increment of the parameters posed a need for floating-point operation per second (FLOPs). The key advantage of half-UNet is the reduction of number of filters during convolution process. As a result of this, during decoder feature fusion, there is a unification in the number of channels in all the feature maps. This is because 3 × 3 convolution is not required by the decoder. This can be seen in the Figure 21, where all the decoder layers are eradicated after receiving input from bottleneck utilizing the input via skip-connection. This is a unique example of a single-stack decoder. As a result, half-UNet becomes a simpler structure. Full-scale feature fusion is the second key component of half-UNet. Keep in mind that concatenation operations are used by both the original UNet and UNet3+ to fuse features.

Although concatenation operation is a fantastic option because it gives better results, its complexity grows because it requires more memory and time. He et al. [269] introduced a ResNet that use addition operation as a feature fusion technique. The authors incorporated their outputs to the stacked layer’s outputs and carried out identity mapping in this procedure. Although more information was added to each dimension, the image’s dimensions were not enlarged by this process. The complexity of the system was not increased by this procedure because there were fewer parameters.

As depicted in Figure 21, this concept is applied in half-UNet. The ⨁ symbol appears, indicating that the skip connections have been fused together to form a single decoder. Reducing convolution complexity is the main principle behind this. As we aware of, the largest computational expense in deep convolutional neural network (DCNN) [269,270,271] is due to convolution operation. There is still significant amount of memory and floating point operations (FLOPS) due to remaining 1 × 1 convolution layer, despite recent works like MobileNet [272,273] and ShuffleNet [274] introducing depth-wise convolution or shuffle operation to build efficient CNNs using smaller convolution filters (floating number operations). The main advantage of the ghost module is to reduce the computational complexity and ability to provide more features via feature maps. The model facilitates in calculation of the parameters and FLOPs during the convolution operation:

(A4) $$parameters=\left(k^{2}*Din+1\right)*Dout$$

(A5) $$FLOPS=2*k^{2}*Din*Dout*HEout*WIout$$

Note that Din and Dout represents input and output sizes, while HEout and WIout represents the height and width of the output maps. Note that * represents the arithmetic products, where the kernel size is represented by k. A ghost module was suggested by Han et al. [275] as a way to produce additional feature maps with inexpensive operations.

In the ghost module, one half of the feature map is generated by the fundamental convolution operation while the second half of the feature map is generated by depth wise separable convolution. These both are then concatenated to form the final output whose dimension is same as the input dimension.

(A6) $$params=\left(k^{2}*\left(Din+1\right)+2\right)*\frac{Dout}{2}$$

(A7) $$FLOPS=2*k^{2}*\left(Din+1\right)*\frac{Dout}{2}*HEout*WIout$$

In the event that the image size is 128 × 128 with 3 × 3 convolution where the number of input and output channels are 64. Using these dimensions, we achieve the number of parameters and FLOPS are 36.92 K and 12.08 G, respectively. However, if the ghost module is used, many parameters are needed, there would only be 18.78 K and 0.61 G FLOPs. As such, Half-UNet makes use of the ghost module.

Advantage and Application of Half-UNet: As we already aware that UNet variants enhanced model performance without changing the design of the U-shaped model. Half-UNet simplifies both the encoder and the decoder. Half-UNet makes use of the ghost module, full-scale feature fusion, and unification of channel numbers. The authors obtained identical segmentation accuracy results when comparing the half-UNet findings with UNet and its variants. Yet the parameters and FLOPs were decreased by 98.6% and 81.8%, respectively, in contrast to UNet. The authors conducted a comparison between the outcomes of half-UNet and UNet, along with its variations, in various medical picture segmentations, such as non-vascular mammography segmentation, non-vascular computed tomography image segmentation of lung nodules, and non-vascular left ventricle MRI image segmentation.

## Appendix F. Bias Assessment

The foundation of the RBM theory is the notion that, in any unbalanced system, a leak will always occur. This leak will always bleed and propagate in a specific direction, leading to a protrusion. When comparing the leak’s strength to the area that hasn’t leaked, it is apparent. The suggested system is made up of AI and its properties, which, when extended in all directions, form a map. A dent, or so-called bias, is created in the system when certain features are excessively strong while others are weak. Because the AI traits span 360 radial directions, This system is what we refer to as a “radial bias” map (RBM). Furthermore, it is possible to represent the regional bias area (RBA), an indirect measure of bias, as the map area that is between the maximum strengths of the AI attributes that the system (study) can contains and the least strengths of the AI traits. To get a cumulative score for each study, we modify the scores for each attribute and each study in the third bias category. After ranking these scores, an estimate of the bias cut-off is made. The “rank-bias” score (RBS) approach is a process for identifying bias that makes use of this kind of ranking paradigm [34].

It has previously been standardized that the role of clusters of attributes plays a vital role in bias estimation. Recently in [34], hybrid deep learning (HDL) technology was demonstrated for COVID diagnosis. These cluster attributes were a representation of the design, optimization strategies for the AI algorithms, performance evaluation of the diagnosis system, and clinical evaluation. In [34], these clusters were 14, 7, 8, and 10 attributes respectively.

We adopt the “spokes and wheel model (SWM)”, for pictorial representation of the strength of the AI traits. The SWM model represents in 360 direction spanning all the spokes of the attributes of the clusters. For a better understanding of the bias estimation algorithm, we follow the same symbols as depicted in [34]. The algorithm can be summarized as follows. As usual first we divide the traits into four prominent clusters, namely, design, optimization, performance evaluation, and clinical validation. The second step consists of spoke length computation of each AI attributes which is mathematically given as a product of attribute weight times the 80% of half of the image size (256). In step three, we compute the sum of the spoke length for each cluster, which is represented as ΣC1, ΣC2, ΣC3, and ΣC4. The fourth step consists of computing the sum of top two and bottom two cluster ΣA and ΣB. In fifth step, we compute the βradial = |ΣA − ΣB| as the absolute difference between ΣA and ΣB, “−” represents subtraction. Finally, we compute the normalized bias value ($\beta_{radial}^{norm}$) =$\;(\frac{\beta radial}{\alpha })$, where α represents the total number of AI traits. An important depiction is the angle between the spokes which is represented as 360/34 = ~10.5 degrees. Finally, to obtain the smooth curve, we use Bezier spline curve to fit to the end point of each spoke. When the spline fits the end of the spokes for the four cluster, the shape represents like a butterfly having four wings as depicted in Figure 17, expended in a 4 × 15 grid, representing 60 experiments.
