# Supplementary material for: Deep Learning Paradigm and Its Bias for Coronary Artery Wall Segmentation in Intravascular Ultrasound Scans: A Closer Look
Source: J Cardiovasc Dev Dis. 2023 Dec 4;10(12):485. doi: 10.3390/jcdd10120485 (PMC10743870; doi:10.3390/jcdd10120485)
Supplement: Supplementary file 1 [file jcdd-10-00485-s001.zip › jcdd-2553559-supplementary.pdf]

Supplementary Materials

Table S1. Non-UNet/conventional method of DL systems.

	Author/Year	Non-UNet Methods	Basic Top-Level Description
1	Bajaj et al. [76] (2021)	Bi-GRU (bidirectional gated recurrent unit)	This study proposed a deep learning approach for automatically identifying end-diastolic frames in intravascular ultrasound pictures. A network model with a bidirectional Bi-GRU structure was trained to detect end-diastolic frames using a DL technique that makes use of variations in the intensity of associated pixels in subsequent NIRS-IVUS frames [76].
2	Vercio et al. [65] (2019)	SVM, RF	Using support vector machines (SVMs), the lumen, media, adventitia, and surrounding tissues were automatically recognized. The type of structure found in each region of the image affected the classification performance of the SVMs. Different morphological structures were discovered using random forest (RF), and the initial layer categorization was changed in accordance with the discovered structure. The LI and MA interfaces were detected using the segmentation approach based on deformable contours using the generated classification maps [65].
3	Cho et al [75] (2021)	Efficient-Net	This study presented IVUS-based deep learning for plaque classification in CAD. For developing the degree-wise classifier, they employed Efficient-Net, which is a kind of neural network search engine designed to maximize not only throughput but also inference efficiency [75] .
4	Araki et al. [43] (2015)	FCM, K-means, HMRF	In this study, the calcium volume was automatically measured based on the boundaries of the coronary artery wall for each frame of the intravascular ultrasonography (IVUS) image. For the automatic segmentation of calcium areas and volume computation, three soft computing fuzzy classification techniques—fuzzy c-means (FCM), k-means, and hidden Markov random field (HMRF)—were used [43].
5	Bajaj et al. [74] (2021)	Pix2Pix model, GAN	The goal of this study was to create and verify a deep learning (DL) methodology that can segment intravascular ultrasonography (IVUS) image sequences automatically and accurately in real time. The ResNet architecture-based convolutional neural network used in the proposed segmentation methodology was a DL model that made use of the Pix2Pix conditional generative adversarial network (GAN) [74].
6	Fedewa et al. [77] (2021)	RF, NIRS-AI	This study examined the possible applications of artificial intelligence (AI) in intracoronary imaging technologies as well as its current and future uses. The use of AI in intracoronary imaging has a lot of potential to advance our knowledge of and ability to treat coronary heart disease [77].
7	Masuda et al. [78] (2021)	CNN	This study focused on image classifications such as the distinction of malignant tumors and calcified coronary plaque through deep learning techniques that demonstrated great diagnostic performance. To estimate coronary plaques, this study compared radiologists' diagnostic performance with that of deep learning using a convolutional neural network (CNN) [78].
8	Min et al. [79] (2021)	XGBoost	The purpose of this study was to create models for stent under expansion prediction based on intravascular ultrasound (IVUS) data collected before to the procedure. A regression model with a convolutional neural network was developed. Binary classification models (XGBoost) were created to distinguish between the frames with and those without under expansion [79].
9	Nishi et al. [80]	CNN, DeepLabv3	In addition to lumen and vascular area, the goal of this study was to establish a DL approach for automatic IVUS segmentation of the stent area. A fully convolutional neural network (CNN) with encoder/decoder networks and the main body served as the foundation for the neural network architecture [80].
10	Shin et al. [111] (2021)	LATM, SATM	This study focused on comparing the reference standard evaluation using intravascular ultrasound (IVUS) imaging with the lumen parameters that were evaluated by the location adaptive threshold method (LATM), in which the inter- and intra-scan impedance variabilities of coronary computed tomographic angiography (CCTA) were addressed, and the scan-adaptive threshold method (SATM), in which only the inter-scan variability was addressed [111].
**11**	Olender et al. [81] (2019)	CNN	They offered a technique for image-based tissue characterization that can be used post hoc to evaluate diseased arteries across complete acquisition sequences. The pixel-based technique used just gray-scale IVUS pictures, convolutional neural networks, and domain knowledge of arterial pathology and physiology to categorize damaged arterial walls into similar tissue categories as virtual histology [81].
12	Zhao et al. [82] (2018)	SVM/RF	They suggested a framework for the automatic multi-class categorization and plaque detection of coronary atherosclerosis. First, they took the computed tomography angiography's transverse cross-sections along centerlines. Second,

			using coarse segmentation, they extracted the region of interest. Thirdly, they retrieved a random radius symmetry (RRS) feature vector, which significantly expanded the training data by combining various descriptions into a random method. Finally, they provided the multi-class coronary plaque classifier, the RRS feature vector [82].
13	Bargsten et al. [83] (2021)	Capsule network	By methodically examining various capsule network architectural variations, they were able to improve the efficiency of IVUS picture segmentation. Then, using different numbers of training images and network settings, they compared their capsule network against similar CNNs [83].
14	Samuel et al. [96] (2020)	VSSC Net	This study focused on early vascular problems that can be identified using the VSSC Net, which separates blood vessels from coronary angiography and retinal fundus pictures [96].
15	Faraji et al. [28] (2018)	Ellipse-fitting algorithm	This study was based on region detection strategy. It had two objectives. First, they examined the viability of defining the luminal– and media–adventitia borders in IVUS frames obtained by 20 MHz probes using the recently proposed extremal area of extremum level (EREL) region detector. Second, based on the stability of their textural information, they suggested a region selection technique to designate two ERELs as lumen and medium [28].
16	Tayel et al. [84] (2017)	Lucy Richardson algorithm, contour initialization	In this study, a method for identifying media–adventitia and lumen borders in intravascular (IV) images was presented. The lumen and media–adventitia contours were calculated using the proposed method using a modified fast algorithm. Fully tested, the improved fast algorithm demonstrated speed increases of up to 200% while maintaining an accuracy rate of 98% [84].
17	Cui et al. [85] (2020)	Gradient boosting	In this study, they created a supervised machine learning technique with excellent accuracy and little user involvement for coronary artery lumen segmentation from IVUS pictures. The fully discriminative lumen segmentation technique was intended to teach both the features of the classifier and a classifier that our weak learners may use. The applied gradient boosting framework's theoretical underpinnings and quadratic approximation were also provided [85].
18	Harms et al. [86] (2021)	RCNN	The suggested method made use of a regional convolutional neural network (RCNN) with a mask-scoring function composed of five main subnetworks: the backbone, the regional proposal network (RPN), the RCNN head, the mask head, and the mask-scoring head. Through the backbone network, multiscale feature maps were learned from computed tomography (CT) [86].
19	Mishra et al. [87] (2018)	FCNN/CNN	The network was subjected to extensive supervision based on the input-output properties of the layers, which enhanced the segmentation accuracy overall. The main contribution of the work is sub-problem-specific deep supervision for ultrasound picture segmentation. The network is currently being tested and trained for inputs of a fixed size. Its performance is diminished in tiny size photos and necessitates image scaling [87].
20	Lin et al. [97] (2022)	ConvLSTM	A novel deep learning approach for quick and automatic plaque quantification from CCTA was created and externally validated by this study. In a large cohort of patients with stable chest discomfort, deep-learning-based plaque and stenosis assessments could predict the likelihood of a future myocardial infarction [97].
21	Du et al. [98] (2022)	4-cascaded RefineNet, DeepLabv3+	This study compared the IVUS segmentation abilities of various convolutional networks and analyzed their effects on a sizable dataset from many regions. When it came to segmenting the lumen contour and EEL on IVUS pictures, the 4-cascaded RefineNet and DeepLabv3 + performed better than UNet and IVUS-net. The most effective segmentation method was DeepLabv3 + [98].
22	Jin et al. [95] (2022)	CNN	With high computational efficiency, the coronary artery was segmented to determine the plaque, and the image patch was extracted using a convolutional neural network (CNN) model. Plaque patterns were collected using the manually created radiomics feature extractor, which was then used to classify the plaques and grade the degree of stenosis [95].
23	Hwang et al. [120] (2018)	Hybrid ensemble classifier	This study's goal was to suggest a hybrid ensemble classifier for identifying locations of coronary plaque in intravascular ultrasonography (IVUS) images. Then, for plaque characterization in this study, a hybrid ensemble classifier based on histogram and texture information was employed. This ensemble classifier's input was the ideal feature set [120].
24	Jodas et al. [88] (2017)	K-means algorithm	This paper presented a fully automatic method for the segmentation of the coronary artery lumen in IVUS pictures. The region corresponding to the possible lumen was identified by the suggested method using the mean roundness and the k-means algorithm. Additionally, a method for locating and removing side branches at bifurcations was suggested to define the area containing the prospective lumen regions [88].
25	Eslamizadeh et al. [89] (2017)	Fuzzy approach	In this study, a fuzzy method was used to segment lumen boundaries at intravascular ultrasound images. The median filter was used to reduce picture noise in

			order to diagnose lumen boundaries during IVUS, and spatial filters in polar coordinates were also used to locate and remove the catheter [89].
26	Sofian/Jodas et al. [90] (2015)	Otsu thresholding	The media adventitia portion of the artery serves as the outside boundary of the artery, and this research presented a method for categorizing and identifying it using intravascular ultrasonography (IVUS) images [90].
27	Cao et al. [91] (2021)	DeepLab V3+	This study showed a fully automatic IVUS multiparameter extraction procedure is suggested to help the cardiologist automatically acquire more clinically important value parameters. They suggested a targeted noise-reduction preprocessing architecture designed for IVUS based on the intima and adventitia obtained by DeepLab V3+ [91].
28	Taki et al. [92] (2008)	Parametric deformable models, geometric deformable models	Two approaches for automatically identifying the media adventitia and intima boundaries in IVUS pictures of the coronary arteries were designed and compared. While the second approach was based on geometric deformable design, the first strategy made use of parametric deformable models. The analysis demonstrated that for automated segmentation of IVUS coronary artery pictures, the geometric deformable model performed better than the parametric deformable model [92].
29	Unal et al. [93] (2008)	Nonparametric intensity model	This study offered a shape-driven method for segmenting the artery wall from rectangular intravascular ultrasound images. They used a nonparametric intensity model based on an image likelihood intensity energy to segment the lumen contour as opposed to the pointwise observations of earlier techniques [93].
30	Zhu et al. [94] (2011)	Gradient vector flow (GVF), snake model	In this paper, a brand-new segmentation technique based on the gradient vector flow (GVF) snake model was suggested. The suggested method's key features were divided into two categories: first, nonlinear filtering was used to the GVF field to decrease the crucial spots, alter the morphology of the parallel curves, and increase the area of capture; second, snake balloons were incorporated into the design [94].

Table S2. UNet method of deep learning systems.

**SN.**	**Author/Year**	**UNet**	**Basic Top-Level Description**
**1.**	Shinohara et al. [62] (2021)	UNet	This study focused on IVUS pictures from patients with angina pectoris that were manually separated into the following categories for AI development using UNet: lumen area, medial plus plaque area, calcification, and stent. Except for stents in IVUS images of complex lesions, the artificial intelligence program correctly identified vessels requiring treatment and vessel components [62].
2.	Ibtehaz et al. [45] (2020)	UNet	They suggested certain adjustments to the UNet model, which is already cutting-edge. After making these adjustments, they created a brand-new architecture called MultiResUNet as a possible replacement for the UNet architecture. On an extensive library of multimodal medical images, they evaluated MultiResUNet and contrasted it with the traditional UNet [45].
3.	Balakrishna et al. [46] (2018)	UNet VGG16	They suggested building a segmentation network using the UNet and a VGG16 encoder. The proposed segmentation architecture was put to the test through experiments, and the results are both quantitatively and qualitatively encouraging [46].
4.	Yang et al. [55] (2019)	Dual-path UNet	This study was about automatically segmenting the lumen and media–adventitia in IVUS frames. This information is paramount in the diagnosis of many CVDs and also makes it easier to create 3D reconstructions of human arteries [55].
5.	Milletari et al. [53] (2016)	VNetFCNN	In this paper, the authors proposed a volumetric, fully convolutional neural network-based method for 3D picture segmentation. The CNN learned to predict segmentation for the entire volume at once after being trained end-to-end on MRI data showing the prostate [53].
6.	Szarski et al. [54] (2021)	UNet	They offered a multi-class fully convolutional semantic segmentation network built on a basic UNet architecture and enhanced with polar translation dependencies. The model could utilize relative spatial context regarding the interior and exterior vessel walls, which are easily separable in polar coordinates thanks to the coordinate awareness in the multiclass segmentation [54].
7.	Xia et al. [71] (2020)	MFAUNet	They suggested using the multi-scale feature-aggregated UNet (MFAUNet) to simultaneously extract the borders of two membranes. The bi-directional convolutional long short-term memory (BConvLSTM) unit, deep supervision, and multiscale inputs were all combined in the MFAUNet. It was intended to learn enough features from challenging IVUS images using a limited number of training examples [71].
8.	Azad et al. [72] (2019)	BCDUNet	For medical image segmentation, they presented an enhancement of UNet, bi-directional ConvLSTM UNet with densely connected convolutions (BCDUNet), in which they fully exploited UNet, bi-directional ConvLSTM (BConvLSTM), and the mechanism of dense convolutions [72].

9.	Zhou et al. [52] (2020)	UNet	This research proposed UNet++, a new neural design for semantic and instance segmentation. It includes reducing unknown network details, reorganizing the skip connections, and developing a pruning scheme [52].
10.	Kim et al. [48] (2018)	UNet multiscale layer	Using intravascular ultrasound (IVUS) images, they suggested a fully convolutional neural network in this research to effectively define the borders of the wall and lumen of the coronary arteries. This network simultaneously addressed multi-label segmentation of the wall and lumen sections [48].
11.	Li et al. [47] (2021)	UNet Deep CNN	In this study, a media–adventitia border, luminal area, and calcified plaque detection end-to-end deep-learning convolutional neural network was built. The three modified UNets were used in the model's construction, together with the idea of cascaded networks, to avoid inaccuracies in the detection of calcification caused by interference from pixels outside the plaque zones [47].
12.	Chen et al. [49] (2019)	3D UNet	In this paper, a fully autonomous 3D multi-channel UNet architecture for coronary artery reconstruction from CTA was proposed. The primary concept of the suggested method was to include the vessels map as the input of the UNet, which acts as the reinforcing information to highlight the tubular structure of coronary arteries, in addition to using the original CTA image [49].
13.	Morris et al. [51] (2019)	3D UNet	This study focused on cutting-edge cardiac substructure segmentation needing a single, non-contrast CT input; they created a revolutionary deep learning (DL) pipeline exploiting MRI's soft tissue contrast combined with CT [51].
14.	Tong et al. [50] (2018)	3D deeply supervised UNet	By combining the multimodal MRI and CT data, this study provided a deeply supervised 3D UNet for fully automatic whole-heart segmentation. To begin, a 3D UNet was used to broadly detect the entire heart and partition its region of interest, which can lessen the impact of neighboring tissues [50].
15.	Song et al. [24] (2021)	3D UNet	In this study, they presented a deep convolutional neural-network-based automatic approach for coronary computed tomography angiography (CCTA) image coronary artery segmentation [24].
16.	Pan et al. [70] (2021)	3D dense UNet	In this paper, a UNet-based network architecture called 3D dense UNet was used to segment the coronary artery completely automatically. This study demonstrated the high-performance detection of coronary lumen vessels made possible by an autonomous segmentation tool, offering the potential capacity to support clinical diagnosis [70].
17.	Dong et al. [61] (2021)	Eight-layer UNet	The cross-sectional area of the coronary artery lumen and the area restricted by the external elastic membrane (EEM) were segmented in this paper using a fully automatic method using an eight-layer UNet (EEM-CSA) [61].
18.	Cheung et al. [60] (2021)	2D UNet	To segment the aorta and coronary arteries on CTCA pictures, authors suggested using a fully automatic two-dimensional UNet model. The aorta and the coronary arteries along with coronary arteries were the regions of interest that the two models were trained to segment [60].
19.	Hwang et al. [69] (2021)	UNet	In this article, authors described an autonomous approach for identifying a vessel's end from X-ray coronary angiography (XCA) that is deep-learning-based. They employed a UNet, which consists of a ResNet encoder and a decoder, for the deep learning network [69].
20.	Thuy et al. [68] (2021)	UNet	Based on the primary and secondary coronary arteries, authors suggested a unique segmenting technique for extracting the coronary arteries from angiograms. For extracting coronary arteries of various diameters, they used a planned coarse-to-fine strategy. Authors developed the first UNet model to separate the main coronary artery extraction and developed a new approach to identify the points where the main coronary artery and secondary coronary artery converge [68].
21.	Shi et al. [64] (2020)	UENET	This research proposed the use of modified generative adversarial networks (GANs) to complete the conversion of a coronary angiography image into a semantic segmentation image. They employed a modified UNet as the generator and a special three-layer pyramid structure as the discriminator [64].
22	Jun et al. [100] (2020)	T-Net	In this study, they proposed the T-Net, a nested encoder decoder architecture. For each block that makes up a convolutional network in T-Net, there are several tiny encoder decoders. The constraint that UNet can have single concatenate layer between the encoder and decoder block is overridden by T-Net [100].
23	He et al. [44] (2020)	Deep attention UNet	They proposed a 3D deep attention UNet model to automatically segment the EAT (epicardial adipose tissue) from CCTA [44].
24	Huang et al. [67] (2022)	UNet	In order to segment the lumen contour in IVOCT images, they suggested in this research a deep residual segmentation network of multi-scale feature fusion based on attention mechanism (RSM-Network, residual squeezed multi-scale network) [67].
25	Jun et al. [59] (2020)	3D UNet	Using only CCTA images, the goal of this work was to create a region-based deep learning technique to automatically detect and segment the LVM. To segment LVM contours from CCTA, they created a 3D deeply supervised UNet that uses attention gates (AGs) to concentrate on the myocardial border structures [59].

26	Momin et al. [58] (2022)	Retina UNet	In this study, a fresh mutual boosting deep learning (DL)-based segmentation method was presented for precise and automatic segmentation, particularly of tiny substructures like coronary arteries. The retina UNet, the classification module, and the segmentation module are the three subnetworks that make up the suggested methodology [58].
27	Javorszky et al. [57] (2022)	Attention UNet	This paper showed that a more accurate prediction of cardiac events is possible with volumetric measurement of coronary artery disease (CAD). However, CAD segmentation requires a lot of labor. The goal was to develop a deep learning (DL) model that is open-source and can be used to segment coronary plaques on coronary CT angiography (CCTA) [57].
28	Shen et al. [56] (2019)	3D FCN	In this study, they jointly presented a level-set approach and deep-learning-based framework for coronary CTA segmentation. The 3D semantic properties of coronary arteries were learned using a 3D fully convolutional network (FCN), which makes a great starting point for the conventional level set. In order to strengthen the vessels and suppress unimportant regions, an attention gate was additionally added to the entire network [56].
29	Ronneberger et al. [73] (2015)	UNet	In this study, authors offered a network and training technique that made heavy use of data augmentation to make better use of the given annotated samples. The architecture comprises a symmetric expanding path that permits exact localization and a contracting path to capture context [73].
30	Yang et al. [66] (2018)	IVUS-Net	In this paper, with a fully convolutional network (FCN), authors solved a key issue in IVUS image analysis: the automatic delineation of the lumen and media–adventitia borders in IVUS pictures, which is essential to speed up diagnosis or facilitate a quicker and more precise 3D reconstruction of the artery [66].
